# Automated RANO 2.0-Aligned Response Assessment for Glioblastoma Clinical Trials: External Evaluation Against Expert Annotations

**DOI:** 10.3390/cancers18203383

**Published:** 2026-10-19

**Authors:** Loizos Siakallis, Sotirios Bisdas

**Affiliations:** 1 Department of Translational Neuroscience and Stroke, UCL Queen Square Institute of Neurology, University College London, Queen Square, London WC1N 3BG, UK; 2 School of Medicine, European University Cyprus, 1516 Nicosia, Cyprus

**Keywords:** glioblastoma, RANO 2.0, response assessment, clinical trials, magnetic resonance imaging, longitudinal imaging, algorithm, neuro-oncology, tumour burden

## Abstract

Background/Objectives: Reliable and reproducible imaging-response assessment is essential for endpoint evaluation in glioblastoma clinical trials. The Response Assessment in Neuro-Oncology (RANO) criteria provide a rule-based framework for standardisation, but their implementation can still yield variable outputs between readers and across timepoints and trial sites. This partly reflects differences in lesion definition, measurement geometry, longitudinal tracking, and new-lesion adjudication. We externally evaluated an automated pipeline for auditable, RANO 2.0-aligned imaging-only response assessment using pre-existing segmentation masks. Methods: This retrospective feasibility and pilot evaluation used longitudinal glioblastoma magnetic resonance imaging (MRI) studies from the public single-centre LUMIERE dataset. Dataset-provided tumour segmentation masks were retained unchanged. An expert neuroradiologist visually reviewed the masks against the corresponding MRI images, and timepoints with segmentations clearly non-representative of tumour tissue were excluded. Deterministic rule-based processing performed lesion definition, admissibility-filtered measurement extraction, longitudinal tracking, new-lesion detection, and provisional response classification. Expert RANO imaging annotations and bidimensional measurements served as comparators rather than complete clinical RANO 2.0 adjudications. Analyses assessed agreement in the sum of products of diameters (SPD), longitudinal burden change, response classification, progression timing, new-lesion agreement, and failure modes, with patient-level clustered bootstrap resampling. Results: Visual segmentation quality control excluded 57 timepoints across 42 patients, leaving 542 evaluable timepoints, including 506 matched to expert annotations. Across 84 expert-measured timepoints from 47 patients, the constrained maximum product of perpendicular diameters (Max-PPD-c) correlated with expert SPD (Pearson r = 0.753; Spearman ρ = 0.861), although the concordance correlation coefficient (CCC) was 0.680 and the median absolute percentage difference was 36.8%. Overall, 73.8% of measurements were within ±50% of expert SPD. Across 31 consecutive pairs from 17 patients, correlations for longitudinal percentage change were r = 0.715 and ρ = 0.874. Per-timepoint four-class response agreement was 48.1% (κ = 0.173), whereas final-timepoint binary progressive disease (PD) agreement was 76.0%. Among patients with PD by both methods, the pipeline assigned PD earlier more often than later, although the median timing difference was 0.0 weeks and the paired comparison was not statistically significant (*p* = 0.068). New-lesion detection had a positive predictive value (PPV) of 22.3% and negative predictive value (NPV) of 89.0%. Conservative patient-level later-positivity estimates were 10.8% within 12 weeks and 27.0% within 24 weeks, without same-lesion verification. Conclusions: This study supports the value of a deterministic pipeline for standardising imaging-derived response outputs in glioblastoma clinical trials. Future work will focus on prospective multi-institutional validation incorporating clinical variables, non-enhancing disease assessment, and adjudicated outcomes.

## Simple Summary

Assessment of treatment response in glioblastoma clinical trials requires reliable and reproducible imaging-based evaluation. However, manual application of response criteria is susceptible to inter-reader variability and can be time-consuming. This study presents an automated pipeline that applies imaging-response criteria to pre-existing segmentation masks. It defines lesions, selects measurable tumour regions, and calculates two-dimensional and volumetric tumour burden. It also tracks lesions over time, flags new lesions, and assigns provisional RANO 2.0-aligned imaging-only response categories. Preliminary evaluation in a public glioblastoma dataset demonstrated moderate-to-strong correlations with expert tumour-burden measurements and longitudinal burden change, although individual measurement differences remained substantial. Categorical response agreement and new-lesion concordance were more limited, highlighting the importance of expert interpretation. The pipeline appears promising for standardisation in research and clinical-trial review. It provides transparent and auditable decision support while maintaining expert oversight. Future work will focus on prospective multi-institutional validation and integration of clinical variables.

## 1. Introduction

Patients with glioblastoma undergo repeated magnetic resonance imaging (MRI) during treatment and follow-up, and imaging-based response assessment is central to clinical-trial endpoint adjudication [1,2]. Although MRI also informs routine clinical decision-making, formal RANO-based response categories are most established in the clinical-trial setting, where reproducible assessment across patients, timepoints, readers, and sites is essential. Despite decades of refinement, radiographic response assessment remains one of the most technically challenging components of neuro-oncology. This is due to factors confounding tumour assessment, which include treatment-related changes, heterogeneous enhancement patterns, necrosis, postoperative cavities, and non-enhancing disease. These complexities limit the reproducibility of conventional size-based criteria and contribute to variability in response classification across observers and institutions [3,4,5]. Standardised response assessment is relevant to trials of emerging neuro-oncology treatments [6,7].

The Response Assessment in Neuro-Oncology (RANO) criteria were developed to standardise response assessment in neuro-oncology trials and to address limitations of earlier frameworks such as the Macdonald criteria [8,9], incorporating both enhancing and non-enhancing disease and accounting for treatment-related effects [10]. RANO 2.0 further unifies response assessment across glioma grades and treatment settings and is intended to support standardised endpoint evaluation in glioma clinical trials. To this end, it retains bidimensional measurements as the primary standard while allowing volumetric alternatives and formalising baseline definition, confirmation rules, and the role of clinical variables [6]. However, as emphasised in operational guidance, reliable implementation of RANO 2.0 requires consistent lesion definition, measurement reproducibility, handling of new lesions, and integration of corticosteroid use and neurological status [11]. In practice, these requirements make trial response assessment time-consuming, observer-dependent, and difficult to standardise.

Recent advances in artificial intelligence (AI) and deep learning have enabled automated tumour segmentation and quantitative burden estimation from MRI. These developments have been enabled by benchmark datasets, self-configuring segmentation frameworks, and large collaborative learning efforts [12,13,14]. Systems such as AutoRANO have demonstrated the feasibility of fully automated bidimensional and volumetric tumour measurements [15,16,17,18], while multicentre studies have shown that automated MRI-derived metrics can correlate with clinical outcomes and survival [16]. More recent work has extended these approaches to longitudinal assessment and response classification. For example, AI-based volumetric tumour response assessment (AI-VTRA) mapped segmentation-derived volumetric changes to response categories and demonstrated moderate agreement with radiologist assessments, although human interpretation remained superior for survival stratification [19].

Despite these advances, most existing approaches focus on tumour segmentation and burden quantification rather than the full execution of response criteria. A key unresolved challenge for trial deployment is how segmentation outputs are translated into RANO-aligned response categories. This includes how lesions are defined in three dimensions, how disconnected tumour foci are merged or separated, how measurement slices are selected, how tumour burden is quantified (2D vs. 3D), how lesion identity is maintained across timepoints, and how new or reappeared lesions are incorporated into response logic. These design choices are not trivial; they directly influence downstream response categorisation and affect how automated tools benchmark against expert readers and trial endpoints.

Evidence from longitudinal studies highlights the importance of these processes. Automated segmentation tools have shown promise for volumetric trend analysis but remain limited by lesion-counting inconsistencies and longitudinal instability, often requiring expert correction [17,20,21]. A recent systematic review identified limited, heterogeneous evidence for automated longitudinal response assessment, with substantial risk of bias and a need for prospective validation against current criteria [21]. In parallel, AI-RANO recommendations emphasise that future systems must prioritise standardisation, interpretability, and clinically trustworthy validation rather than focusing solely on predictive performance [22,23].

Another emerging distinction is between reader-facing tools and backend analytical frameworks. Structured response-reporting and reader-facing tools, including BT-RADS and RANO2.0-assist, illustrate this adjacent but distinct category of support systems [24,25,26,27]. However, such tools are primarily designed to assist clinical reading rather than to systematically evaluate alternative lesion definitions, measurement geometries, and longitudinal rules to enable response classification.

In this context, there remains a gap for a reproducible and auditable pipeline that explicitly implements the steps linking segmentation outputs to imaging-only response assessment. Addressing this gap is particularly relevant for glioblastoma clinical trials and central imaging review, where response criteria require consistent implementation.

In this feasibility and pilot evaluation study, we present an automated pipeline for RANO 2.0-aligned longitudinal imaging-only response assessment in glioblastoma clinical trials. The pipeline uses longitudinal MRI and dataset-provided segmentation masks to define three-dimensional lesion entities, applies admissibility-filtered measurements, tracks lesions across timepoints, and generates provisional, auditable response outputs for expert review. We evaluated the pipeline using the LUMIERE dataset, which provides longitudinal glioblastoma MRI with expert RANO annotations [28]. Specifically, we performed a structured evaluation comprising internal method consistency, external technical concordance with expert measurements, longitudinal trend analysis, progression-timing assessment, and failure-mode characterisation.

The aim of this study is to evaluate whether a configurable automated pipeline can support reproducible and standardised RANO-aligned response assessment while enabling transparent and auditable integration with expert human review. By isolating and analysing the steps between segmentation and response classification, this work seeks to identify methodological factors that influence agreement with expert interpretation and trial-facing response outputs. The pipeline is intended to support consistent longitudinal assessment in clinical-trial and central-review settings and provide a foundation for future clinical decision-support systems following prospective validation.

## 2. Materials and Methods

### 2.1. Study Design and Analytical Framework

This retrospective proof-of-concept external evaluation applied a deterministic automated pipeline for RANO 2.0-aligned imaging-only response assessment to longitudinal glioblastoma MRI from the public LUMIERE dataset. In this manuscript, “framework” describes the broader methodological approach to automated response assessment. “Pipeline” refers to the implemented processing downstream of tumour segmentation evaluated in this study. No de novo segmentation model was trained or validated. Dataset-provided DeepBraTumIA-derived masks were retained unchanged to preserve the public benchmark. They were used to isolate the effects of lesion definition, measurement geometry, longitudinal tracking, new-lesion logic, and response-rule execution. Although the broader framework includes segmentation and the use of alternative automated or manually corrected masks, these options were not used in the primary analysis. The pipeline translated the unchanged masks into lesion entities, bidimensional and volumetric tumour-burden measures, TrackID-based longitudinal lesion states, new-lesion flags, and provisional response categories. The analysis did not incorporate clinical variables such as corticosteroid exposure, neurological status, treatment context, and clinical deterioration. Also, imaging analysis did not incorporate T2/FLAIR progression or pseudoprogression windows.

Analyses followed four domains: (i) internal consistency between automated measurement geometries using fixed rules for lesion definition, slice selection, tracking and response classification; (ii) technical concordance with expert sum of products of diameters (SPD) and RANO categories; (iii) longitudinal burden-change and time-to-first-progression behaviour; and (iv) lesion-count concordance, new-lesion agreement, and discordance patterns.

Max-PPD-c was the primary automated bidimensional burden metric, implemented as the constrained automated analogue of RANO-style calliper measurement. It required both diameters to remain within contiguous enhancing tumour on an admissible slice. PPD, Feret, and Max-PPD were evaluated exploratorily to quantify the effect of measurement geometry. Supplementary results comprise an overall performance summary (Table S1), measurement-method comparisons (Table S2), temporal new-lesion analyses (Tables S3 and S4), longitudinal burden-threshold comparisons (Table S5), quality-control comparisons (Table S6), response-rule analyses (Table S7), response confusion matrices (Table S8) and observed discrepancies (Table S9).

### 2.2. LUMIERE Dataset and Cohort Definition

#### 2.2.1. Source Dataset and Expert RANO Comparator

The external analysis used the public LUMIERE dataset, a single-centre longitudinal glioblastoma MRI cohort with expert RANO evaluation comprising 91 patients, 638 study dates, and 2487 MRI images. Skull-stripped NIfTI MRI and co-registered DeepBraTumIA-derived tumour masks provided standard structural sequences and labels for enhancing tumour, necrosis, and oedema. These masks were treated as fixed source inputs and were not corrected, replaced, or regenerated by the present pipeline.

Expert RANO annotations included complete response (CR), partial response (PR), stable disease (SD), and progressive disease (PD) categories, lesion-level classifications, and bidimensional measurements where available. Expert annotations were treated as an independent comparator rather than a multi-reader clinical reference standard. Pipeline records were matched to annotations by exact patient identifier and study date after duplicate patient-timepoint key resolution, and only unique exact matches entered matched analyses. Expert LUMIERE annotations were used as imaging-derived RANO CR/PR/SD/PD response comparators for pipeline response-classification analyses, whereas bidimensional measurements were compared separately in continuous SPD concordance analyses; these annotations did not constitute full clinical RANO 2.0 adjudications.

#### 2.2.2. Inclusion, Exclusion, and Evaluability Criteria

Native LUMIERE images and dataset-provided segmentation masks were analysed without reprocessing, manual correction, or re-segmentation. An expert neuroradiologist performed timepoint-level segmentation quality control (QC) by visually reviewing the dataset-provided masks against the corresponding MRI images. Masks were excluded only when clearly non-representative of tumour tissue, including segmentation of subdural collections, choroid plexus, vascular structures, postoperative changes, extra-axial structures, or other non-tumour anatomy. Automated–expert measurement disagreement alone was not an exclusion criterion. No mask was edited, and other valid examinations from the same patient were retained. Representative examples of segmentation quality-control exclusions are provided in Figure S1.

Quality-control exclusions were applied at the timepoint level, with other valid examinations from the same patient retained. Endpoint-specific evaluability was defined after quality control, exact matching, and availability of the required expert measurement or response annotation; continuous SPD analyses required expert bidimensional measurements, whereas response, new-lesion, and timing analyses used the relevant expert annotations. Performance before and after QC was compared to assess the effect of exclusions.

### 2.3. RANO-Aligned Longitudinal Imaging-Response Pipeline

#### 2.3.1. Input Data and Preprocessing Checks

The automated pipeline was used for imaging-only longitudinal response assessment. The pipeline ingested serial MRI examinations and corresponding dataset-provided tumour segmentation masks in NIfTI format after spatial-compatibility, voxel-dimension and label-definition checks. Contrast-enhancing tumour (label 1) was the primary target label. Non-enhancing/necrotic tissue (label 2) was used as an exclusion label for measurement-slice admissibility, while label 3 represented oedema. No separate postoperative-cavity segmentation was generated. Dataset preprocessing and segmentation preceded the present evaluation, and source images and masks were retained without modification.

#### 2.3.2. Lesion Definition and Connectivity Rules

The pipeline defines lesion entities in three dimensions prior to any measurement. Lesion definition employs 3D connectivity-based separation of enhancing tumour masks, with distance-based grouping of spatially related disconnected foci when applicable. The 10 mm target-size threshold was based on the RANO minimum-size requirement for both perpendicular diameters of measurable disease [6]. The 5 mm separation distance provided a consistent spatial rule for grouping nearby disconnected foci. Lesion measurements informed target-lesion eligibility, and all measurement methods used the same lesion-definition rules within each examination so that comparisons were not confounded by changes in lesion inputs. Optional graph-based clustering was not activated in the present analysis.

#### 2.3.3. Target-Lesion Selection and Admissibility Criteria

The pipeline assigns lesion roles according to enhancing-tumour measurability rules, and target lesions meeting minimum-size criteria contribute to timepoint-level burden. In the current analysis, candidate measurement slices were excluded when more than 5% of the measurement region consisted of non-enhancing necrotic or cavity label. This implementation setting was intended to keep measurement regions predominantly within enhancing tumours. Among admissible slices, the selected slice maximised the method-specific measurement objective. Target-lesion selection, slice admissibility, longitudinal ordering, and aggregation rules were fixed across measurement methods.

The pipeline can also process non-enhancing T2/FLAIR labels and select lesions under FLAIR-based configurations. These options were not activated in the primary LUMIERE analysis, which used enhancing-tumour targets to match the available expert bidimensional comparator; independent non-enhancing progression was therefore not evaluated.

#### 2.3.4. Tumour-Burden Quantification (Bidimensional and Volumetric)

For each lesion, the pipeline derives both bidimensional and volumetric representations of tumour burden. Classical axis-aligned perpendicular product (PPD) and Feret-derived measurements (based on calliper diameters across the lesion contour) are computed using the same lesion-definition and slice-selection rules. Additionally, an orientation-optimised maximum product of perpendicular diameters (Max-PPD) is calculated, defined as the maximum product obtained under unrestricted orientation optimisation. A constrained variant (Max-PPD-c), in which both diameters are required to lie entirely within contiguous enhancing tumour (within-lesion chord constraint) while respecting slice-admissibility criteria, is also implemented. Bidimensional products are reported in mm^2^. Volumetric burden is calculated as the number of enhancing target-lesion voxels multiplied by voxel volume and is reported in mm^3^. At each timepoint, tumour burden is defined as the sum of the selected target-lesion measurements for each method. This allows comparison of alternative measurement geometries using shared lesion inputs and aggregation rules. Volume provides a three-dimensional comparator and is not an SPD-equivalent measurement. Representative examples of automated Max-PPD-c measurement placement and objective-driven slice selection are provided in Figure S2.

#### 2.3.5. Longitudinal Lesion Tracking and State Assignment

The longitudinal module was designed to maintain lesion identity through the propagation of unique deterministic lesion identifiers (“TrackIDs”) across ordered examinations. Baseline lesions are assigned unique identifiers, and follow-up lesions are matched using a hierarchical correspondence strategy based on spatial overlap and distance. Unmatched lesions are classified as new, while lesions absent at an intervening time point but subsequently re-matched to a prior TrackID are classified as reappeared. These lesion states are incorporated into downstream response classification.

For response classification, the first post-operative MRI available in each patient’s LUMIERE series was selected as the analytical baseline. Although we aimed for consistency with the RANO 2.0 definition of baseline, the post-radiotherapy time point was not provided within the dataset. CR was defined as zero current target burden, including when baseline burden was zero; PR as a ≥50% reduction in bidimensional burden or ≥65% reduction in volumetric burden relative to a positive baseline burden; PD as a ≥25% increase in bidimensional burden or ≥40% increase in volumetric burden relative to a positive baseline burden or a positive prior nadir, or positive current target burden when baseline burden was zero; and SD otherwise. PD was assigned whenever the pipeline flagged a new enhancing lesion, overriding response categories based on target-lesion measurements. Otherwise, CR was assigned when the summed measurements of the selected target lesions were zero.

Detailed connectivity, slice-selection, measurement, tracking, and audit specifications are provided in the Supplementary Material. These outputs were interpreted as provisional imaging-only response categories, rather than validated clinical RANO 2.0 adjudications. The implemented baseline and tracking rules were retained in the analysis of response-rule dependence.

### 2.4. Internal Method Consistency and Method Comparison

Internal consistency was evaluated within the fixed primary configuration. At each evaluable timepoint, response categories were compared pairwise across PPD, Feret, Max-PPD, Max-PPD-c, and volumetric burden using four-class CR/PR/SD/PD agreement, unweighted Cohen’s κ, and secondary binary PD versus non-PD agreement.

Continuous bidimensional burden behaviour was compared pairwise using Pearson correlation, Spearman correlation, and symmetric absolute percentage difference. Volume was retained as a contextual three-dimensional comparator and was not treated as an SPD-equivalent measure.

Exploratory method comparison in the refined comparison cohort used expert SPD as reference and summarised Pearson and Spearman correlation, concordance correlation coefficient (CCC), median absolute percentage difference, tolerance-band agreement and Bland–Altman limits-of-agreement width. Individual metrics were interpreted descriptively without an overall ordinal ranking. Max-PPD-c was retained as the primary reporting metric.

### 2.5. External Concordance with Expert Imaging Annotations

#### 2.5.1. Continuous Agreement with Expert SPD

The primary technical endpoint was the matched patient-timepoint agreement between automated Max-PPD-c SPD and expert-derived SPD. Association was described using Pearson and Spearman correlation. Numerical agreement was assessed using the concordance correlation coefficient, median absolute percentage difference from expert SPD, tolerance-band agreement relative to expert SPD (±25%, ±50%, and ±100%), and Bland–Altman bias and limits of agreement. This endpoint evaluated technical measurement concordance rather than clinical diagnostic accuracy, outcome prediction, or interchangeability. Exploratory comparisons repeated these analyses for PPD, Feret, and Max-PPD under identical matching, quality-control, and cohort definitions.

Per-timepoint four-class agreement between expert RANO imaging categories and pipeline-derived provisional imaging-only response categories at matched examinations was quantified using exact observed agreement and unweighted Cohen’s κ.

#### 2.5.2. Binary PD vs. Non-PD

For binary progression analyses, response categories were collapsed to PD versus non-PD (CR, PR, or SD). Using expert imaging-derived RANO annotations as a comparator, agreement was evaluated with accuracy, sensitivity, specificity, positive predictive value (PPV), negative predictive value (NPV), and unweighted Cohen’s κ.

The final-timepoint agreement was assessed at each patient’s last matched evaluable examination for four-class and binary endpoints using exact observed agreement. These patient-level summaries complemented per-timepoint analyses affected by repeated visits and class imbalance. An exploratory sensitivity analysis compared agreement with expert response categories with and without the new-lesion PD override. Categories were calculated from the saved target-lesion measurements, baseline burden, prior nadir and new-lesion flags. The remaining response rules were retained. Comparisons used the same 318 evaluable examinations and the same 75 selected final visits, without reprocessing MRI (Table S7).

#### 2.5.3. Expert Versus Automated New-Lesion Agreement

The agreement between expert-reported new lesions and the automated new-enhancing-lesion flag was assessed at the matched patient-timepoint level. Expert new-lesion status was derived from structured and text-based RANO annotations, whereas the automated flag identified enhancing components classified as new by the longitudinal module under the selected overlap threshold. Metrics included observed agreement, unweighted Cohen’s κ, concordance counts, and comparator-based sensitivity, specificity, PPV, and NPV. Because expert annotations were imaging comparators rather than final clinical adjudications, these metrics were interpreted descriptively.

#### 2.5.4. Exploratory Temporal Cross-Confirmation of Discordant New-Lesion Calls

To assess whether same-timepoint new-lesion discordance reflected timing differences, we performed an exploratory bidirectional temporal cross-confirmation analysis. For each patient and direction, we identified the first discordant event: pipeline-positive/expert-negative or expert-positive/pipeline-negative, excluding events in which the confirming method had previously been positive. Subsequent positivity by the other method was assessed at the next available follow-up, within 12 weeks, within 24 weeks, and at any later follow-up from the discordant index call. Direction-specific and combined temporal cross-confirmation analyses are reported in Table S3, whereas conservative all-index temporal confirmation analyses are reported in Table S4. Because expert and automated lesion identifiers were not shared, confirmation was interpreted as patient-level temporal concordance rather than lesion-level verification.

### 2.6. Longitudinal Trend and Progression-Timing Analyses

Longitudinal agreement was assessed among consecutive expert-measured timepoints within the same patient. Percentage change in tumour burden was calculated between consecutive matched examinations for each automated method and the expert SPD.

#### 2.6.1. Longitudinal Burden-Change Agreement

Longitudinal comparisons assessed: (i) correlation of percentage change using Pearson and Spearman coefficients; (ii) directional agreement, classifying changes as increase, decrease, or stable; and (iii) burden-threshold agreement, applying RANO-like thresholds for worsening (≥25% increase) and improvement (≥50% decrease). Changes between these thresholds were classified as stable. Threshold disagreement was defined as different burden-change categories assigned by expert and automated measurements for the same consecutive examination pair. Pairs with undefined percentage change because of zero previous burden were excluded from this calculation. This analysis assessed consecutive-pair burden changes rather than complete RANO response categories based on baseline, nadir, new lesions and clinical information.

Analyses were performed using the matched expert-measured timepoint pairs within the analysis cohort.

#### 2.6.2. Time-to-Progression and Paired Timing Analysis

Time-to-progression was the first matched timepoint at which PD was assigned independently by expert RANO assessment and by pipeline-derived provisional response. For each patient with at least one PD event, the first-PD week was identified separately for the expert and pipeline series. For patients with PD assigned by both methods, Δweek was defined as pipeline first-PD week minus expert first-PD week; negative values indicated earlier pipeline PD assignment.

Timing summaries included the numbers of patients with earlier, identical, or later pipeline PD assignment and the number without PD by one or both methods. Paired Δweek values were compared using the Wilcoxon signed-rank test. Analyses were performed for all patients with PD by both methods and for the subgroup with concordant final PD classification.

### 2.7. Discordance and Failure-Mode Analyses

Sources of expert–pipeline disagreement were examined using predefined row-level and summary outputs. Analyses used matched examinations to compare automated and expert SPD measurements, lesion counts, and new-lesion status. Lesion-count concordance was based on agreement in the number of enhancing target lesions identified at matched examinations, whereas new-lesion concordance was based on agreement between expert-reported and pipeline-flagged new enhancing lesions.

Discordance domains were: (i) automated-versus-expert SPD differences; (ii) target-lesion selection or lesion-count disagreement; (iii) new-lesion disagreement; (iv) segmentation-related implausible burden distributions; and (v) factors outside the imaging-only response assessment. The analysis included examination of lesion counts, new-lesion flags and longitudinal matching outputs. These findings were not treated as mutually exclusive causes of disagreement. The relative contributions of registration, segmentation, postsurgical change and lesion splitting or merging could not be established from these outputs alone. Observed discrepancies and longitudinal matching findings are summarised in Table S9.

### 2.8. Statistical Analysis

All analyses were descriptive, performed within predefined analytic cohorts, and conducted without imputation or adjustment for multiple comparisons. Absolute percentage difference was calculated relative to expert SPD; symmetric absolute percentage difference was calculated as 200 × |x − y|/(x + y); and Bland–Altman limits of agreement were calculated as mean difference ±1.96 SD.

Uncertainty estimates used patient-level clustered bootstrap resampling with 2000 iterations. Patients were sampled with replacement as the resampling unit; all eligible timepoints from each sampled patient were retained, and all reported metrics were recalculated within each replicate, including correlations, percentage-difference summaries, tolerance-band proportions, Bland–Altman statistics, κ statistics, binary classification metrics, timing-category proportions, and paired Δweek summaries. Two-sided 95% confidence intervals (CIs) used percentile empirical bootstrap distributions, thereby reflecting variability at the patient level rather than treating repeated examinations as independent observations. Exploratory linear and quadratic weighted Cohen’s κ supplemented the primary unweighted κ. Categories were ordered as CR, PR, SD and PD, indexed 0–3. Linear disagreement weights were |i − j|/3 and quadratic weights were (i − j)^2^/9. Confidence intervals used 2000 patient-level clustered percentile bootstrap replicates.

For time-to-progression analyses, bootstrap resampling preserved expert-pipeline pairing within each patient. Δweek comparisons used the Wilcoxon signed-rank test. Exploratory temporal new-lesion confirmation rates were summarised among window-evaluable discordant events; conservative all-index proportions were reported in the Supplementary Material. Confidence intervals used patient-level percentile bootstrap resampling. Analyses were performed using IBM SPSS Statistics (version 31.0.2.0; IBM Corp., Armonk, NY, USA) [29] and Python (version 3.12) [30] with standard scientific libraries. Because the evaluated pipeline was deterministic and involved no AI model training, optimisation, or inference, CLAIM and STARD-AI were not applied as reporting frameworks. The manuscript nevertheless follows the principle that software, parameters, data sources, and validation limitations should be reported transparently for medical-imaging algorithms [31]. During manuscript preparation, OpenAI ChatGPT (GPT-5.5) was used for language editing, structural refinement and editorial suggestions. The authors reviewed and edited all AI-assisted output.

## 3. Results

### 3.1. Cohort Flow, Matching Yield, and Quality Control

After duplicate-key resolution, 599 unique pipeline timepoints and 6065 lesion-level records were analysed; 563 timepoints matched a unique expert RANO assessment, and 141 had expert bidimensional measurements before quality control. Prespecified blinded neuroradiologist QC excluded 57 timepoints across 42 patients without editing source images or masks, yielding 542 evaluable timepoints, 506 expert-matched timepoints, 84 paired expert-measured timepoints, and 75 patients with sufficient longitudinal data for response and progression-timing analyses. The response-classification cohort comprised 318 matched examinations with paired expert and pipeline categorical response outputs after quality-control and endpoint-specific evaluability filtering. All 91 patients retained at least one pipeline timepoint, with 42 patients having partial exclusions. For the SPD comparison, 15 patients lost all available paired measurements and 27 had partial exclusions. For response classification, four patients lost all evaluable observations and 38 had partial exclusions. Endpoint-specific exclusions and performance before and after QC are reported in Table S6. In the post-QC cohort of 542 examinations from 91 patients, the median number of examinations per patient was 5 (IQR 3–8, range 1–19). The corresponding median was 1 in the SPD subset of 47 patients (IQR 1–2, range 1–10) and 3 in the response-classification subset of 75 patients (IQR 2–6, range 1–14).

Table 1 summarises cohort definition, matching yield, and quality-control filtering. Figure S3 summarises the cohort flow and endpoint-specific analysis populations.

Analyses used the largest eligible denominator for each endpoint. Endpoint-specific analysis populations were therefore used for SPD agreement, response classification, new-lesion agreement, longitudinal burden change, and progression-timing analyses. This reflected differential availability of expert bidimensional measurements relative to expert CR/PR/SD/PD response annotations within the LUMIERE dataset.

### 3.2. Internal Consistency of Pipeline Measurement Methods

Across 318 evaluable timepoints, all automated measurement methods evaluated for response classification, including volumetric burden, produced identical categorical responses: pairwise CR/PR/SD/PD agreement was 100%, with Cohen’s κ = 1.00 for both multiclass and binary PD versus non-PD analyses. The response-rule analysis showed that all 185 PD assignments were determined by the shared new-lesion override. Of the 133 CR assignments, 101 occurred when positive baseline target-lesion burden decreased to zero, whereas 32 occurred when target-lesion burden was zero at both baseline and follow-up. These CR assignments were based on target-lesion measurements and did not establish disappearance of all enhancing disease. The identical response categories across measurement methods therefore reflected the shared new-lesion and zero-target-burden rules, rather than demonstrating that differences between measurement methods had no effect on response classification (Table S7).

Continuous target-lesion burden estimates were method-dependent: PPD and Feret showed Pearson r = 0.986, Spearman ρ = 0.975, and median symmetric absolute percentage difference 7.9%, whereas Max-PPD-c comparisons with classical methods had lower linear correlation and median symmetric differences of 14.6% to 20.2%.

Continuous consistency analyses were restricted to bidimensional SPD-based methods. Volume remained a contextual three-dimensional comparator rather than an SPD-equivalent measure, although computed by the same pipeline and used for response classification.

In exploratory method comparison against expert SPD, Max-PPD-c showed the highest Spearman correlation, the highest proportion of observations within ±50% of expert SPD and the narrowest Bland–Altman limits. Max-PPD had the highest Pearson correlation, whereas Feret had the highest CCC and the lowest median absolute percentage difference. These comparisons remained exploratory.

### 3.3. Pipeline-Expert Quantitative Agreement (SPD)

In the analysis cohort (n = 84 expert-measured timepoints from 47 patients), Max-PPD-c SPD yielded Pearson r = 0.753, Spearman ρ = 0.861, CCC = 0.680 (95% CI 0.575–0.808), median absolute percentage difference 36.8%, and 73.8%/94.0% of observations within ±50%/±100% of expert SPD; confidence intervals are reported in Table S2. After segmentation quality control, no zero automated burden occurred with positive expert measurements.

**Figure 1:**
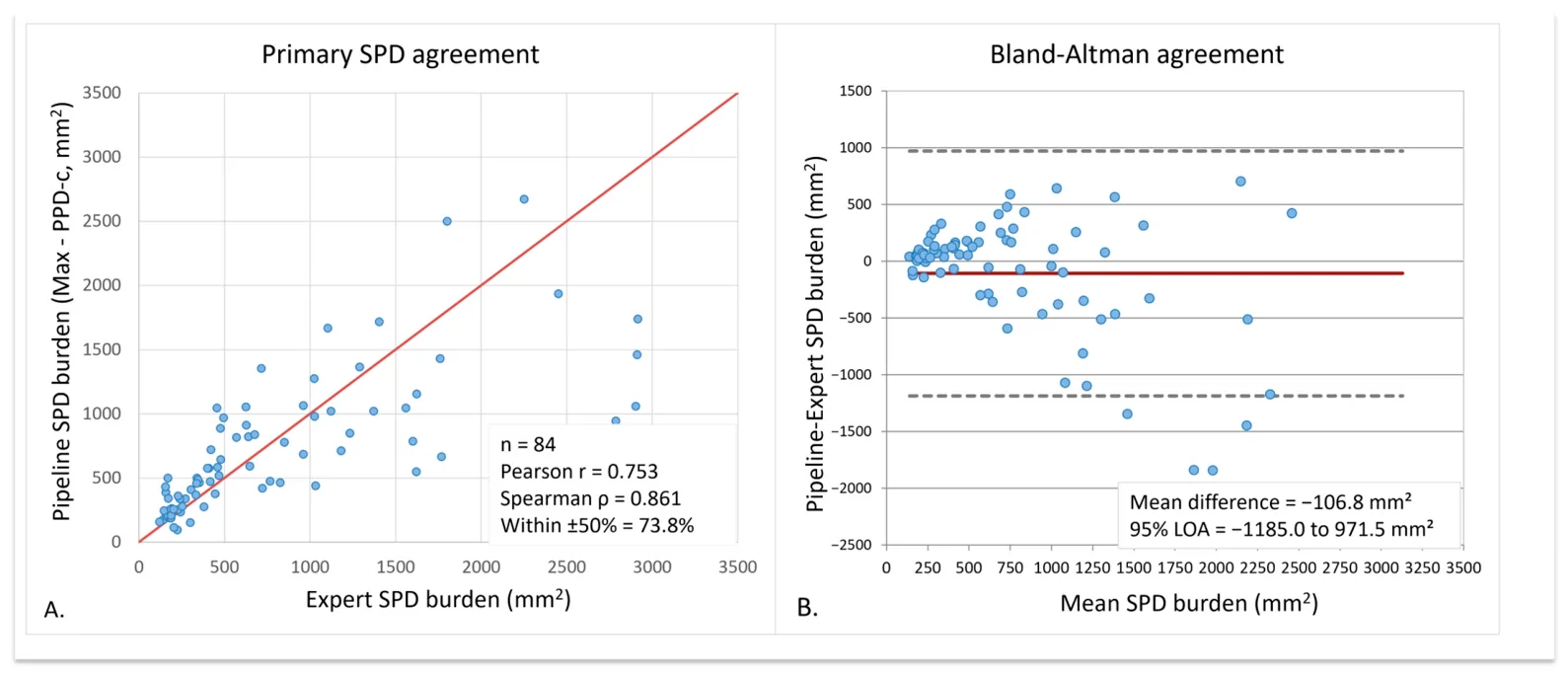
Agreement between expert SPD and automated Max-PPD-c tumour-burden measurement. (**A**) Scatter plot of expert-derived SPD versus automated Max-PPD-c SPD across 84 expert-measured timepoints from 47 patients in the final analysis cohort. The diagonal line indicates identity. Pearson r = 0.753, Spearman ρ = 0.861, and 73.8% of observations were within ±50% of expert SPD. (**B**) Bland–Altman analysis of automated Max-PPD-c versus expert SPD. The *x*-axis shows mean SPD burden and the *y*-axis shows pipeline-minus-expert SPD difference. The solid line indicates the mean difference (−106.8 mm^2^), and dashed lines indicate the 95% limits of agreement (−1185.0 to 971.5 mm^2^). The concordance correlation coefficient was 0.680. SPD = sum of products of diameters.

Exploratory methods demonstrated the highest Pearson correlation for Max-PPD (r = 0.771), the highest Spearman correlation (ρ = 0.861) and ±50% agreement (73.8%) for Max-PPD-c, and the lowest median absolute percentage difference for Feret (29.7%). For Max-PPD-c versus expert SPD, Bland–Altman mean difference was −106.8 mm^2^, with limits of agreement from −1185.0 to 971.5 mm^2^ (Figure 1). Before QC, the SPD comparison included 141 examinations from 62 patients, compared with 84 examinations from 47 patients after QC. For Max-PPD-c, CCC increased from 0.269 to 0.680, while the median absolute percentage difference decreased from 63.5% to 36.8% (Table S6).

### 3.4. Longitudinal Burden Behaviour

Longitudinal analyses included 31 consecutive expert-measured timepoint pairs from 17 patients within the analysis cohort. For the primary Max-PPD-c metric, Pearson correlation for percentage change between consecutive timepoints was 0.715, and Spearman correlation was 0.874. Directional agreement between automated and expert assessments was 77.4%, and burden-threshold agreement was 71.0%.

**Figure 2:**
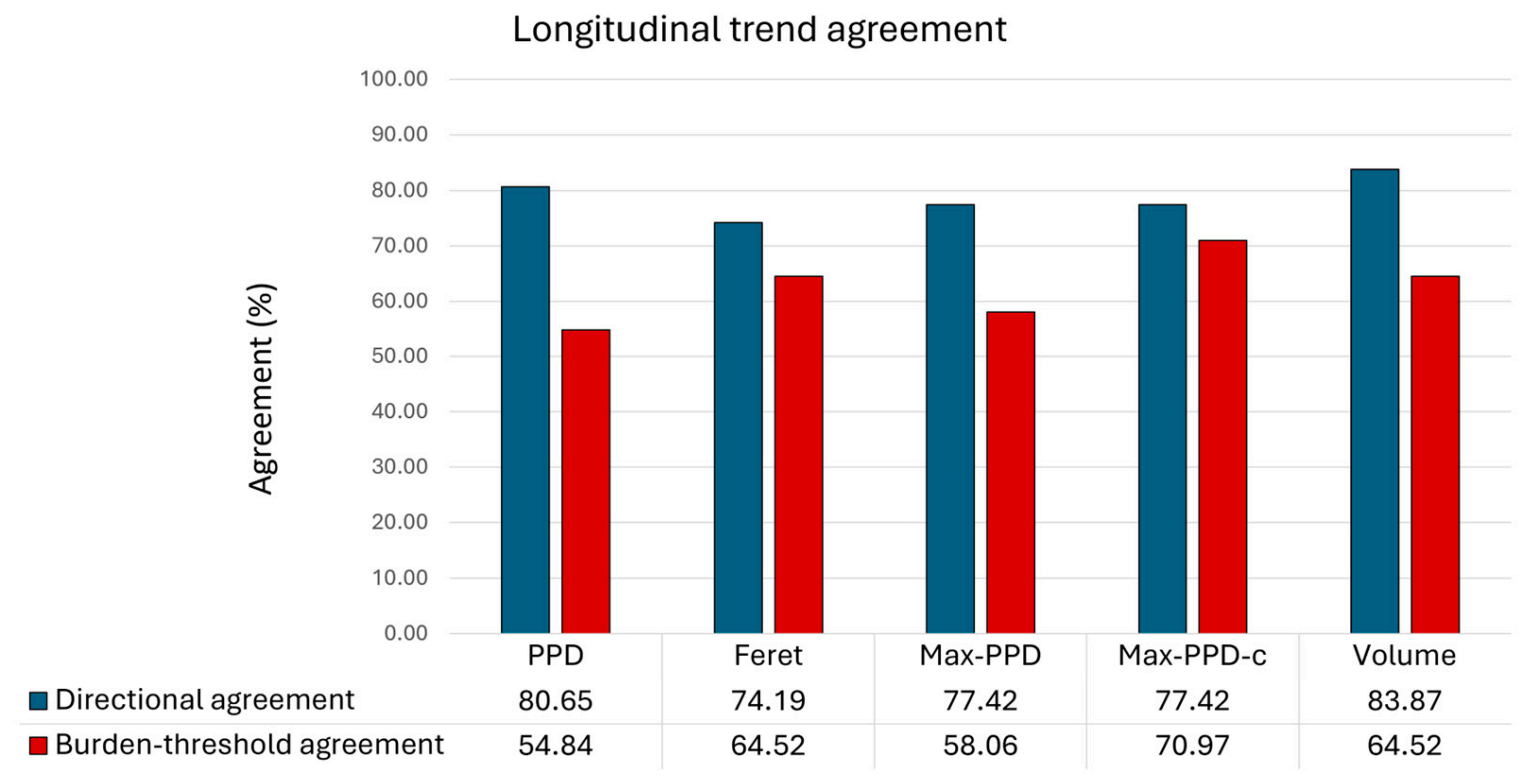
Longitudinal trend agreement across automated measurement methods. Bars show directional agreement and burden-threshold agreement between expert and automated burden changes across 31 consecutive expert-measured examination pairs from 17 patients. Threshold categories describe changes between consecutive examinations rather than complete baseline/nadir-based RANO response categories. For the primary Max-PPD-c method, directional agreement was 77.4% and burden-threshold agreement was 71.0%. Volume is shown as a contextual comparator.

Across the four bidimensional measurement methods, directional agreement ranged from 74.2% to 80.6% and burden-threshold agreement from 54.8% to 71.0%, with Max-PPD-c demonstrating the highest burden-threshold agreement. Burden-threshold categories differed between expert and automated measurements in 14/31 pairs for PPD (45.2%), 11/31 for Feret (35.5%), 13/31 for Max-PPD (41.9%) and 9/31 for Max-PPD-c (29.0%) (Table S5). As a contextual comparator, volumetric burden changes showed 83.9% directional agreement (26/31 pairs) and 64.5% burden-threshold agreement (20/31 pairs) with expert SPD changes (Figure 2).

### 3.5. Imaging-Based Response Classification: Expert RANO Versus Pipeline

Among 318 matched examinations, four-class CR/PR/SD/PD exact agreement between expert RANO and imaging-only pipeline categories was 48.1% (153/318; 95% CI 41.4–54.3%), with unweighted Cohen’s κ = 0.173 (95% CI 0.094–0.252). Exploratory linear weighted κ was 0.225 (95% CI 0.120–0.331), and quadratic weighted κ was 0.270 (95% CI 0.148–0.393) (Table S8). Pipeline outputs were polarised into CR and PD in the post-exclusion response table (133 CR, 185 PD, no PR or SD). The expert distribution was CR 26, PR 19, SD 88 and PD 185; the expert–pipeline confusion matrix is provided in Table S8. These agreement estimates do not establish clinical validity of the provisional imaging-only response categories.

For the binary PD versus non-PD endpoint, observed agreement was 64.2% (204/318, 95% CI 59.2–68.9%), with unweighted Cohen’s κ = 0.263. Using expert annotation as the comparator, the binary PD analysis showed 128 concordant PD classifications, 76 concordant non-PD, 57 pipeline-PD/expert-non-PD, and 57 expert-PD/pipeline-non-PD, yielding sensitivity, specificity, PPV, and NPV of 69.2%, 57.1%, 69.2%, and 57.1%, respectively. These estimates should be interpreted as descriptive clustered concordance measures rather than independent diagnostic-performance estimates.

Final-timepoint analyses were intrinsically patient-level and included 75 patients with evaluable longitudinal response data. Exact final-category agreement was 69.3% (52/75; 95% CI 58.7–78.7%), and binary PD versus non-PD agreement was 76.0% (57/75; 95% CI 66.7–85.3%). Four-class response agreement was 54.1% (203/375, κ = 0.196) before QC and 48.1% (153/318, κ = 0.173) after QC. Comparisons of categorical response, new-lesion agreement and final-timepoint agreement before and after QC are provided in Table S6.

For Max-PPD-c, omitting the new-lesion override changed 123/318 response categories (38.7%). Exact four-class agreement with expert assessment decreased from 153/318 (48.1%, κ = 0.173) to 84/318 (26.4%, κ = 0.087). Of the 165 initially discordant classifications, 57 (34.5%) were PD assignments from the new-lesion override paired with an expert non-PD category. Omitting the override resolved eight disagreements, changed 38 classifications without resolving disagreement, and made 77 initially concordant classifications discordant. At the 75 final visits, 30 categories changed. Four-class agreement decreased from 52/75 (69.3%) to 27/75 (36.0%), and binary PD agreement decreased from 57/75 (76.0%) to 37/75 (49.3%). Detailed results for all measurement methods are provided in Table S7.

At the final visits, expert categories were CR 1, PR 5, SD 7 and PD 62, compared with pipeline categories of CR 17 and PD 58. The full confusion matrices and class distributions are provided in Table S8.

### 3.6. Progression-Timing Analyses

**Figure 3:**
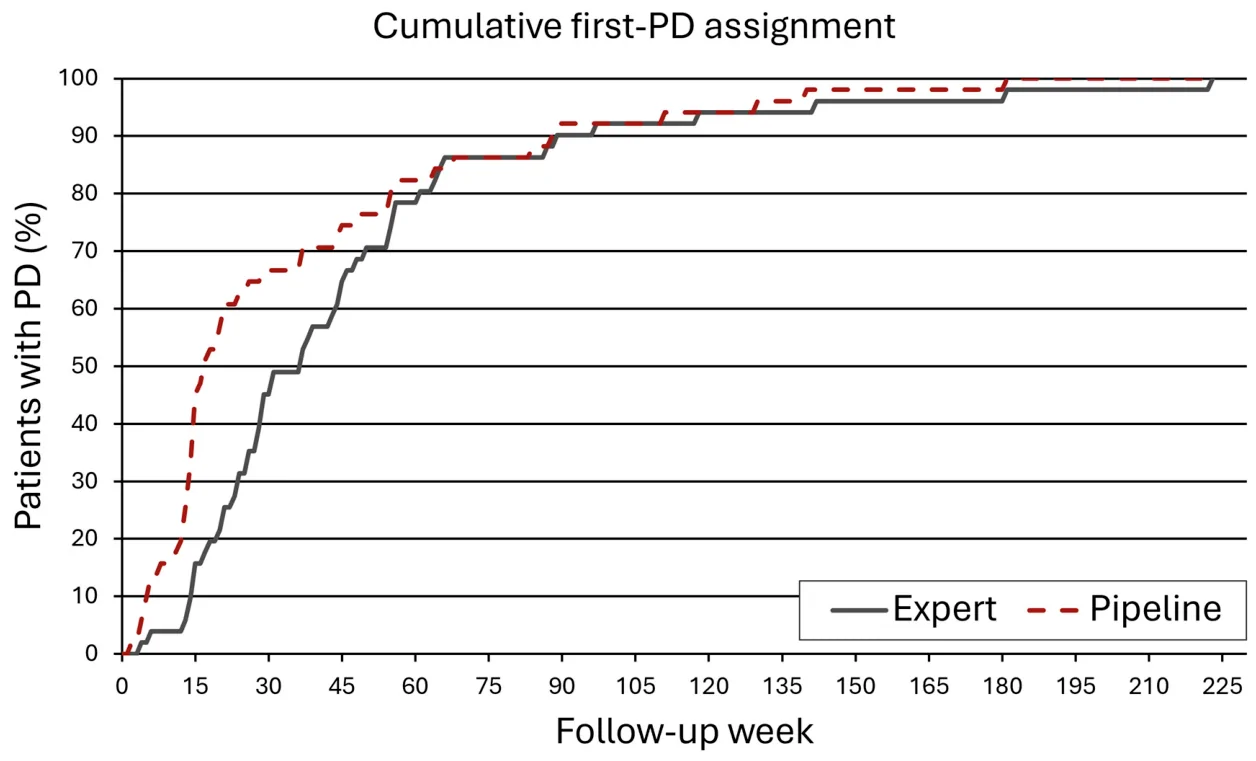
Cumulative first-PD assignment among patients with concordant final PD classification. The plot shows the cumulative proportion of the agreed-final-PD subgroup (n = 51) in whom progressive disease had been called by each follow-up week according to both expert assessment and the pipeline. This figure corresponds to the subgroup with 22 same-week, 20 earlier-pipeline, and 9 later-pipeline first-PD assignments. The curves represent first-PD assignments according to expert imaging annotations and pipeline rules.

Time-to-progression analysis included 75 patients with evaluable longitudinal response data. Among these, first-PD timing was identical in 26 (34.7%), earlier by pipeline in 21 (28.0%), later by pipeline in 11 (14.7%), and absent by one or both methods in 17 (22.7%). Among patients with PD assigned by both methods (n = 58), counts were 21 earlier, 26 identical, and 11 later; median Δweek was 0.0 weeks (interquartile range −17.5 to 0.0), with Wilcoxon signed-rank *p* = 0.068. In the concordant final-PD subgroup (n = 51), 22 patients (43.1%) had identical first-PD timing, 20 (39.2%) earlier pipeline PD, and 9 (17.6%) later pipeline PD; median Δweek was 0.0 weeks (interquartile range −21.0 to 0.0), with *p* = 0.073. Cumulative first-PD assignment in the concordant final-PD subgroup is shown in Figure 3.

### 3.7. New-Lesion Detection Performance

Treating expert RANO imaging annotation as the comparator, sensitivity for expert-reported new lesions was 66.7% (95% CI 52.3–80.3%), and specificity was 53.8% (95% CI 47.1–61.1%). PPV and NPV of the automated new-enhancing-lesion flag were 22.3% (95% CI 15.9–29.6%) and 89.0% (95% CI 84.3–93.5%), respectively. Overall agreement was 55.9%, with Cohen’s κ = 0.114. These values are descriptive comparator-based concordance metrics rather than estimates of clinical diagnostic accuracy.

Concordance counts comprised 56 concordant positive, 227 concordant negative, 195 pipeline-only positive, and 28 expert-only positive new-lesion flags across the analysed timepoints.

Exploratory temporal analysis identified 74 first discordant new-lesion events: 63 pipeline-positive/expert-negative and 11 expert-positive/pipeline-negative. In the combined bidirectional analysis, later positivity by the other method occurred in 17 of 62 window-evaluable events at the next follow-up (27.4%, 95% CI 16.9–39.1), 8 of 24 within 12 weeks (33.3%, 14.3–52.9), 20 of 55 within 24 weeks (36.4%, 23.5–49.2), and 38 of 62 at any later follow-up (61.3%, 49.2–73.2). Directional and conservative all-index temporal analyses are reported in Tables S3 and S4. Using all 74 discordant index events, conservative later-positivity estimates were 8/74 (10.8%) within 12 weeks and 20/74 (27.0%) within 24 weeks. Events without eligible follow-up were considered unconfirmed in this analysis. These findings are hypothesis-generating and describe later positivity by the other method at patient level, without same-lesion verification.

**Figure 4:**
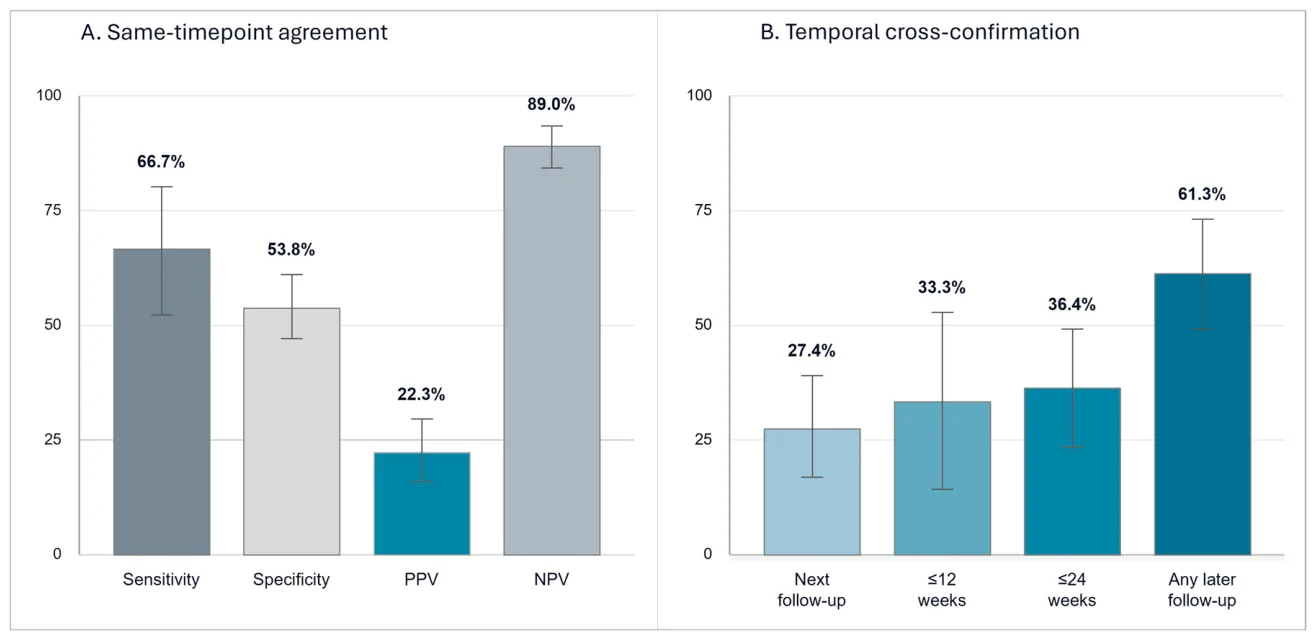
Exact and temporal new-lesion concordance between expert RANO imaging annotations and the automated new-enhancing-lesion flag. Error bars indicate patient-cluster percentile-bootstrap 95% confidence intervals. (**A**) Exact same-timepoint agreement metrics across 506 matched examinations using expert imaging annotation as comparator. Sensitivity was 66.7%, specificity 53.8%, PPV 22.3%, and NPV 89.0%. (**B**) Exploratory combined bidirectional temporal cross-confirmation analysis of discordant new-lesion calls. Cross-confirmation by the other method occurred in 27.4% of evaluable discordant events at the next follow-up, 33.3% within 12 weeks, 36.4% within 24 weeks, and 61.3% at any later follow-up. Temporal analyses combine both discordance directions and use window-evaluable denominators. They represent patient-level concordance rather than lesion-level verification. Conservative estimates based on all discordant index events are reported in Table 2 and Table S4. PPV = positive predictive value; NPV = negative predictive value.

The results for new-lesion agreement for the same-timepoint and patient-level temporal cross-confirmation analyses are summarised in Table 2 and visualised in Figure 4.

### 3.8. Sources of Discordance and Failure Modes

Differences between automated and expert SPD measurements, lesion counts, and new-lesion status were evaluated using predefined row-level discrepancy flags. In 84 expert-measured timepoints, lesion-count agreement was 75.0%, with pipeline counts higher in 8 cases, lower in 13, and median difference 0. Across 506 matched timepoints, new-lesion concordance counts were 56 concordant positive, 227 concordant negative, 195 pipeline-only positive, and 28 expert-only positive flags. Review of the longitudinal matching outputs identified limitations in the recorded component-level correspondence (Table S9). These findings may contribute to new-lesion disagreement and emphasise the importance of reviewing pipeline-defined new lesions against the corresponding MRI examinations.

Additional retained discordances involved atypical lesion distributions or disproportionate tumour-burden estimates relative to expert measurements. Some response-category differences relative to expert RANO annotations were not explained by imaging-derived burden or lesion-state variables. These may reflect factors outside the scope of the imaging-only pipeline.

### 3.9. Summary of Principal Findings

In summary, the pipeline showed moderate-to-strong correlation with expert SPD and longitudinal percentage change but limited four-class (CR/PR/SD/PD) response concordance and substantial new-lesion discordance. Patient-level final-timepoint binary PD agreement was higher than per-timepoint four-class agreement. These findings support the pipeline in its current state as an auditable imaging-based support tool under human supervision. A consolidated summary of principal quantitative results is provided in Table S1.

## 4. Discussion

The principal contribution of this study is the conception and preliminary evaluation of an auditable automated pipeline for RANO 2.0-aligned imaging-only response assessment of longitudinal glioblastoma MRI in a clinical trial setting. The pipeline translates segmentation masks into lesion entities, admissibility-filtered bidimensional and volumetric measurements, longitudinal lesion tracks, new-lesion flags, and provisional response categories. This approach is clinically important because response assessment in glioblastoma trials depends on how lesion identity, measurement geometry, longitudinal tracking, and response rules are executed. The value of the pipeline therefore lies in standardising and auditing the steps between image-derived tumour burden and trial-facing response outputs. Unlike approaches focused primarily on segmentation, volumetric burden mapping, or reader-assistance, the present evaluation isolates the deterministic processing steps downstream of segmentation. These include lesion definition, admissibility-filtered measurement, longitudinal tracking, new-lesion handling, and response-rule execution.

This positioning is particularly relevant to RANO 2.0. The updated criteria provide a unified framework for glioma response assessment and retain bidimensional measurement as the primary imaging paradigm while allowing volumetric alternatives [6]. Operational guidance further emphasises consistent handling of measurable disease, baseline definition, new lesions, corticosteroid use, neurological status, and treatment context [11]. The present pipeline addresses the imaging-processing component of this framework by explicitly modelling lesion definition, measurement admissibility, longitudinal identity, and rule-based response assignment. As the current implementation did not incorporate clinical parameters, its outputs should be interpreted as imaging-only, provisional RANO-aligned response categories rather than complete clinical RANO 2.0 adjudications. In particular, the assessment did not include corticosteroid exposure, neurological status, treatment context, progression of non-enhancing lesions, or confirmation of progressive disease.

Using LUMIERE as an external evaluation dataset, Max-PPD-c showed moderate-to-strong correlation with expert SPD. However, the CCC of 0.680, the 36.8% median absolute percentage difference, and the wide Bland–Altman limits indicate that the measurements should not be considered interchangeable at individual examinations. Per-timepoint four-class CR/PR/SD/PD agreement was more modest. This divergence is important for clinical-trial application. The threshold analysis also showed that measurement differences could lead to different categories of longitudinal burden change. The 36.8% median absolute percentage difference describes measurement differences at individual examinations and should not itself be interpreted as the proportion of response classifications affected. The released LUMIERE dataset contained expert CR/PR/SD/PD annotations for more examinations than corresponding expert bidimensional measurements. This suggests that some expert categorical assignments incorporated interpretative information not fully reconstructible from the available geometric measurements alone. Nevertheless, correlated tumour-burden measurements do not necessarily produce concordant categorical agreement when thresholds, new-lesion logic, lesion tracking, and additional clinical qualifiers influence the final response label. In this context, imaging-based RANO-aligned assessment should not yet be treated as an autonomous substitute for expert central review when the latter is used as the reference standard.

Patient-level analyses showed higher agreement than per-timepoint analyses. The final-timepoint agreement was higher than the per-timepoint agreement, with 69.3% exact final-category agreement and 76.0% binary final-PD agreement. In the overall paired-PD cohort (n = 58; patients with PD assigned by both methods), the pipeline assigned PD earlier more often than later (36.2% versus 19.0%). Similarly, in the concordant final-PD subgroup (n = 51; patients with concordant final PD classification by both methods), earlier pipeline PD assignment was also more frequent than later assignment (39.2% versus 17.6%). Because Δweek was defined as pipeline first-PD week minus expert first-PD week, negative values indicated earlier pipeline PD assignment. Median Δweek remained 0.0 weeks, and paired testing did not reach statistical significance. Given the low positive predictive value of new-lesion flags relative to expert assessment, earlier pipeline PD assignments may reflect new-lesion disagreement and do not yet establish earlier biological progression or clinical benefit. Future prospective studies should assess whether earlier pipeline PD assignment corresponds to clinically symptomatic earlier progression, progression-free survival, treatment-decision endpoints or overall survival.

Internal method comparison showed complete categorical agreement across automated bidimensional methods, despite measurable differences in continuous tumour-burden estimates. The observed pipeline outputs, however, were limited to CR and PD. This separation between categorical stability and quantitative variability reinforces the importance of response-rule execution. Exploratory comparison against expert SPD also showed that no single bidimensional metric was uniformly superior across all measures. Max-PPD-c had a strong overall profile, including the highest Spearman correlation, the highest proportion of observations within ±50% of expert SPD and the narrowest Bland–Altman limits among the tested methods. However, categorical agreement reflected the shared new-lesion and zero-target-burden rules and was limited to CR and PD, leaving robustness across all four response categories untested. Other methods performed better on specific metrics (for example, Max-PPD showed the highest Pearson correlation, whereas Feret-derived PPD showed the lowest median absolute percentage difference). These findings support Max-PPD-c as the primary analytical metric while showing that measurement geometry remains a non-neutral design choice in automated response assessment.

To assess the contribution of the new-lesion rule override to response-category agreement, we performed a sensitivity analysis. This compared the classifications with and without the override rule while retaining the other response rules. With the new-lesion override, a pipeline PD category could agree with an expert PD even when the expert did not report a new lesion. Removing the override resolved eight disagreements but made 77 previously concordant classifications discordant, reducing four-class agreement from 48.1% to 26.4% for Max-PPD-c. These findings demonstrate that agreement on response category does not necessarily indicate agreement on new-lesion status. The higher agreement with the override does not establish the validity of individual new-lesion flags. The override-off results also remain dependent on segmentation, target selection and baseline/nadir handling and therefore do not represent an independent assessment of measurement geometry.

The study extends prior automated glioma imaging work by moving beyond segmentation and tumour-burden quantification toward execution of longitudinal response logic. Earlier studies demonstrated the feasibility of automated bidimensional and volumetric measurement and the prognostic value of quantitative MRI features [15,16]. More recent response-classification studies have mapped overall volumetric burden change to response categories, but without explicit longitudinal lesion tracking or new-lesion logic [19]. The present pipeline adds configurable lesion identity, lesion definition, longitudinal tracking, and new-lesion handling within a standardised deterministic framework for RANO-aligned measurement and response assessment. Such configurability is important because lesion-definition and lesion-selection rules may vary substantially between readers and studies. The positive predictive value for new lesions relative to expert judgement was 22.3% at the same examination. In exploratory temporal analyses, the other method later reported a new lesion in some patients with an initial disagreement. Conservative estimates of this later positivity were 10.8% within 12 weeks and 27.0% within 24 weeks. These findings did not establish that both methods had identified the same lesion. Residual discordance may reflect segmentation or tracking errors and differences in lesion grouping, particularly around postoperative cavities. Expert annotations were not linked to pipeline components at the lesion level. We therefore did not independently verify, for each discordant call, whether the relevant focus was measurable or non-measurable, whether it was considered a separate new lesion or extension of existing disease, or how its spatial relationship to previous enhancement informed the expert interpretation. These remain potential explanations requiring lesion-level adjudication rather than demonstrated causes of disagreement. Because new-lesion flags override burden-based categories and result in PD, these findings emphasise the importance of expert review of flagged lesions. This is also consistent with previous concerns regarding lesion counting and longitudinal instability in automated longitudinal assessment [20]. At the same time, interobserver variability in glioma response assessment and lesion measurement is well recognised. Equivocal cases in clinical-trial settings may require adjudication or additional expert review. These factors may contribute to uncertainty when using expert assessments as the reference standard [3,11].

The exploratory temporal new-lesion analysis additionally suggested partial later convergence of discordant expert and pipeline calls at the patient level, although lesion-level verification was not possible because expert and automated lesion identifiers were not shared. Because new enhancing lesions overrode burden-based response categories, such discordance probably contributed to the more modest per-timepoint four-class agreement and to earlier pipeline PD assignment in some patients, whereas higher final-timepoint binary PD agreement suggests partial convergence at the patient endpoint level.

In clinical trials, central imaging review and endpoint adjudication require reproducible measurements and transparent rule execution. They also require traceable failure modes across serial examinations. The proposed pipeline provides these functions in an auditable format. It can support sensitivity analyses of lesion definitions, measurement geometries and longitudinal rules. These analyses can assess how alternative configurations affect response classification. This is consistent with AI-RANO recommendations for standardisation and validation. These recommendations also emphasise interpretability and integration into clinical workflows while maintaining expert oversight [22,23]. The near-term implication is support for methodological development, quality control and expert-assisted review in glioblastoma trials. With further development and independent validation, the broader framework may also support future clinical application by structuring tumour-burden trends and complementing expert assessment.

Several limitations should be acknowledged. First, the response module was imaging-only and did not incorporate clinical parameters. These include corticosteroid dose, neurological status, treatment context, clinical deterioration and confirmation rules. Baseline timing relative to radiotherapy completion was not verified within the dataset, and pseudoprogression windows were not applied. This limits interpretation as full clinical RANO 2.0 adjudication, although independent non-enhancing progression adjudication was not a core part of the evaluated imaging-based criteria implementation. Second, the study used a single-centre dataset with expert imaging annotations rather than a multi-reader clinical reference standard. Clustered bootstrap resampling accounted for repeated examinations, but the effective sample size remains patient-limited. The quantitative comparison included 84 examinations from 47 patients, and the longitudinal burden-change analysis included 31 pairs from 17 patients, limiting generalisability. Third, because the present evaluation deliberately used fixed dataset-provided segmentations, segmentation inaccuracies within the LUMIERE dataset may propagate into lesion definition, tracking, new-lesion detection, and response classification. Hence, the results should be interpreted as performance of the deterministic pipeline conditional on the available segmentations, rather than as validation of a complete MRI-to-response workflow. In this context, the broader framework already provides preprocessing and automated segmentation capabilities. However, the present analysis deliberately retained the dataset-provided segmentations unchanged to isolate evaluation of the pipeline downstream of segmentation. Fourth, the pipeline configuration differs from human interpretation: strict slice-admissibility thresholds and fixed spatial rules may split or exclude lesion components that expert readers would handle more flexibly. This is particularly relevant when enhancement is equivocal, adjacent to necrosis or a postoperative cavity, or distributed across spatially related foci. Sensitivity analyses of different configuration parameters were beyond the scope of this comparison of measurement methods and will be addressed in future studies. Such parameters include lesion-separation distance, tracking tolerance, the new-lesion overlap threshold and lesion clustering distances. Finally, volumetric measurements were implemented and incorporated into response classification, but dedicated expert volumetric longitudinal reference standards were not available as comparators. Future studies could compare automated volumetric outputs against manually corrected or expert-refined segmentation-derived volumetric assessments.

The present study evaluated technical performance against expert imaging annotations and did not assess clinical outcomes or effects on treatment decisions. Future work should focus on prospective multi-institutional validation in phases 2 and 3 glioblastoma trial datasets with standardised imaging protocols, harmonised follow-up, and experienced neuroradiologists’ decisions as reference standards [21,31,32]. Integration of clinical variables required for full RANO 2.0 implementation, including corticosteroid use and neurological status, will be necessary before any autonomous RANO adjudication or clinical deployment. Further refinement of segmentation quality control and new-lesion detection is likely essential. Such studies should evaluate not only agreement with expert RANO assessment but also clinically meaningful endpoints. These include progression-free and overall survival, treatment decisions, and patient clinical outcomes, in line with recent calls for prospective validation and standardisation in automated neuro-oncology imaging [21]. Prospective validation of the pipeline could also test whether combined enhancing and non-enhancing lesion quantification improves prediction of clinically meaningful endpoints. This need to complement technical performance with clinical validation is also highlighted in recent studies [33].

## 5. Conclusions

This feasibility and pilot evaluation assessed an auditable deterministic pipeline for automated imaging-only response assessment using existing glioblastoma MRI segmentation masks. The findings reinforce the importance of consistent execution of response criteria downstream of segmentation. The main contribution is a common framework for comparing measurement geometries using shared lesion inputs and tracing measurements and rule-based response outputs through the audit tables. Correlation with expert tumour-burden measurements and longitudinal burden change was moderate-to-strong, although individual measurements were not interchangeable with expert SPD. Categorical response agreement and same-timepoint new-lesion agreement remained limited.

These findings support further evaluation of the pipeline for expert review, quality control and research standardisation. The potential of such analyses to support treatment-response assessment in glioblastoma clinical trials requires prospective validation. Future work will focus on multi-institutional validation, integration of clinical variables required for full RANO 2.0 assessment, evaluation of non-enhancing progression, and assessment against clinically meaningful outcomes.

## Figures and Tables

**Table 1:** Cohort definition, matching, and quality-control filtering.

Analysis Set	*n*	Principal Use
Source LUMIERE dataset	91 patients; 638 study dates; 2487 MRI images	Source cohort
Unique pipeline timepoints after duplicate-key handling	599	Pre-QC pipeline cohort
Lesion-level records	6065	Lesion-level audit outputs
Expert-matched timepoints before QC	563	Pre-QC exact patient-date matching
Expert-measured timepoints before QC	141	Pre-QC bidimensional comparator
QC-excluded timepoints	57 timepoints across 42 patients	Excluded non-representative segmentations
Final post-QC timepoints	542	Overall evaluable pipeline cohort
Expert-matched timepoints after QC	506	New-lesion and categorical comparator analyses
Expert-measured timepoints after QC	84	SPD agreement and lesion-count concordance
Response-classification evaluable matched examinations	318	Four-class and binary response concordance
Consecutive expert-measured pairs	31	Longitudinal burden-change analysis
Longitudinal response cohort	75 patients	Final-timepoint and first-PD analyses

Pipeline-derived timepoints were matched to expert RANO imaging annotations by exact patient identifier and study date. Endpoint-specific denominators reflect visual segmentation QC, evaluability and availability of the relevant expert comparator. The post-QC SPD comparison included 84 examinations from 47 patients, longitudinal burden-change analyses included 31 consecutive examination pairs from 17 patients, and response analyses included 318 examinations from 75 patients. QC = quality control; PD = progressive disease; SPD = sum of products of diameters.

**Table 2:** Exact same-timepoint metrics used expert new-lesion annotation as the comparator. Temporal cross-confirmation used the first discordant event per patient per direction, excluding events in which the confirming method had previously been positive, and pooled both directions. Temporal confirmation indicates later positivity of the other method at patient level, not lesion-level confirmation. Confidence intervals (CIs) are patient-cluster percentile bootstrap intervals. Conservative estimates use all 74 discordant index events, with events lacking eligible follow-up considered unconfirmed. NPV = negative predictive value; PPV = positive predictive value.

Analysis	*n/N*	Result, % (95% CI)
Exact same-timepoint sensitivity	56/84	66.7 (52.3–80.3)
Exact same-timepoint specificity	227/422	53.8 (47.1–61.1)
Exact same-timepoint PPV	56/251	22.3 (15.9–29.6)
Exact same-timepoint NPV	227/255	89.0 (84.3–93.5)
Combined temporal cross-confirmation: next visit	17/62	27.4 (16.9–39.1)
Combined temporal cross-confirmation: ≤12 weeks	8/24	33.3 (14.3–52.9)
Combined temporal cross-confirmation: ≤24 weeks	20/55	36.4 (23.5–49.2)
Combined temporal cross-confirmation: any later follow-up	38/62	61.3 (49.2–73.2)
Combined conservative later positivity: ≤12 weeks	8/74	10.8
Combined conservative later positivity: ≤24 weeks	20/74	27.0

## Data Availability

The imaging data, tumour segmentation masks, and expert RANO annotations analysed in this study are publicly available through the LUMIERE dataset, as described by Suter et al. [28]. Derived aggregate results are reported in this article and its Supplementary Material. Additional derived analysis outputs may be made available by the corresponding author upon reasonable request, subject to institutional approvals and data-use conditions. The pipeline script is identified as rano_34_final.py, with SHA-256 hash db280b52390bc7e3ca09815a7c0f8428b8c82cdb041f33a396d31c6e06ac3d50. The source code has not been publicly released because of ongoing development and intellectual-property considerations. Configuration parameters are reported in the Methods and Supplementary Section S11. The Supplementary Material also describes the measurement, tracking and response rules, while Figure S2 provides a representative example of imaging inputs and measurement outputs. Statistical procedures and analysis software versions are reported in Section 2.8. These details support documentation and auditability, although independent replication of the complete implementation remains limited. We aim to make the code available in a future prospective study, subject to completion of the intellectual-property processes.

## Supplementary Materials

The following supporting information can be downloaded at: https://www.mdpi.com/article/10.3390/cancers18203383/s1. Supplementary Methods, technical specification of the RANO-aligned longitudinal imaging-response pipeline; Figure S1, examples of segmentation quality-control exclusions; Figure S2, example of auditable Max-PPD-c measurement and slice selection; Figure S3, cohort flow and analysis populations; Table S1, summary of automated pipeline performance against expert RANO assessment; Table S2, exploratory comparison of automated bidimensional tumour-burden methods against expert SPD; Table S3, bidirectional temporal cross-confirmation of discordant new-lesion calls; Table S4, conservative all-index temporal confirmation analysis of discordant new-lesion calls; Table S5, longitudinal burden-threshold comparisons; Table S6, performance before and after visual segmentation quality control; Table S7, response-rule analysis and sensitivity to the new-lesion override; Table S8, expert–pipeline response matrices and categorical agreement estimates; Table S9, frequencies of observed discrepancies and matching findings.

## References

[1] Stupp R., Mason W.P., Van Den Bent M.J., Weller M., Fisher B., Taphoorn M.J.B., Belanger K., Brandes A.A., Marosi C., Bogdahn U. (2005). Radiotherapy plus concomitant and adjuvant temozolomide for glioblastoma. N. Engl. J. Med..

[2] Louis D.N., Perry A., Wesseling P., Brat D.J., Cree I.A., Figarella-Branger D., Hawkins C., Ng H.K., Pfister S.M., Reifenberger G. (2021). The 2021 WHO classification of tumors of the central nervous system: A summary. Neuro-Oncol..

[3] Vos M.J., Uitdehaag B.M.J., Barkhof F., Heimans J.J., Baayen H.C., Boogerd W., Castelijns J.A., Elkhuizen P.H.M., Postma T.J. (2003). Interobserver variability in the radiological assessment of response to chemotherapy in glioma. Neurology.

[4] Reardon D.A., Ballman K.V., Buckner J.C., Chang S.M., Ellingson B.M. (2014). Impact of imaging measurements on response assessment in glioblastoma clinical trials. Neuro-Oncol..

[5] Chukwueke U.N., Wen P.Y. (2019). Use of the response assessment in neuro-oncology (RANO) criteria in clinical trials and clinical practice. CNS Oncol..

[6] Wen P.Y., Van Den Bent M., Youssef G., Cloughesy T.F., Ellingson B.M., Weller M., Galanis E., Barboriak D.P., De Groot J., Gilbert M.R. (2023). RANO 2.0: Update to the response assessment in neuro-oncology criteria for high- and low-grade gliomas in adults. J. Clin. Oncol..

[7] Khilar S., Dembinska-Kenner A., Hall H., Syrmos N., Ligarotti G.K.I., Plaha P., Apostolopoulos V., Chibbaro S., Barbagallo G.M.V., Ganau M. (2025). Towards a new dawn for neuro-oncology: Nanomedicine at the service of drug delivery for primary and secondary brain tumours. Brain Sci..

[8] Macdonald D.R., Cascino T.L., Schold S.C., Cairncross J.G. (1990). Response criteria for phase II studies of supratentorial malignant glioma. J. Clin. Oncol..

[9] Ramakrishnan D., Von Reppert M., Krycia M., Sala M., Mueller S., Aneja S., Nabavizadeh A., Galldiks N., Lohmann P., Raji C. (2023). Evolution and implementation of radiographic response criteria in neuro-oncology. Neuro-Oncol. Adv..

[10] Wen P.Y., Macdonald D.R., Reardon D.A., Cloughesy T.F., Sorensen A.G., Galanis E., DeGroot J., Wick W., Gilbert M.R., Lassman A.B. (2010). Updated response assessment criteria for high-grade gliomas: Response assessment in neuro-oncology working group. J. Clin. Oncol..

[11] Ellingson B.M., Sanvito F., Cloughesy T.F., Huang R.Y., Villanueva-Meyer J.E., Pope W.B., Barboriak D.P., Shankar L.K., Smits M., Kaufmann T.J. (2024). A neuroradiologist’s guide to operationalizing the response assessment in neuro-oncology (RANO) criteria version 2.0 for gliomas in adults. AJNR Am. J. Neuroradiol..

[12] Menze B.H., Jakab A., Bauer S., Kalpathy-Cramer J., Farahani K., Kirby J., Burren Y., Porz N., Slotboom J., Wiest R. (2015). The multimodal brain tumor image segmentation benchmark (BRATS). IEEE Trans. Med. Imaging.

[13] Isensee F., Jaeger P.F., Kohl S.A.A., Petersen J., Maier-Hein K.H. (2021). nnU-Net: A self-configuring method for deep learning-based biomedical image segmentation. Nat. Methods.

[14] Pati S., Baid U., Edwards B., Sheller M., Wang S.-H., Reina G.A., Foley P., Gruzdev A., Karkada D., Davatzikos C. (2022). Federated learning enables big data for rare cancer boundary detection. Nat. Commun..

[15] Chang K., Beers A.L., Bai H.X., Brown J.M., Ly K.I., Li X., Senders J.T., Kavouridis V.K., Boaro A., Su C. (2019). Automatic assessment of glioma burden: A deep learning algorithm for fully automated volumetric and bidimensional measurement. Neuro-Oncol..

[16] Kickingereder P., Isensee F., Tursunova I., Petersen J., Neuberger U., Bonekamp D., Brugnara G., Schell M., Kessler T., Foltyn M. (2019). Automated quantitative tumour response assessment of MRI in neuro-oncology with artificial neural networks: A multicentre, retrospective study. Lancet Oncol..

[17] Rudie J.D., Calabrese E., Saluja R., Weiss D., Colby J.B., Cha S., Hess C.P., Rauschecker A.M., Sugrue L.P., Villanueva-Meyer J.E. (2022). Longitudinal assessment of posttreatment diffuse glioma tissue volumes with three-dimensional convolutional neural networks. Radiol. Artif. Intell..

[18] Nalepa J., Kotowski K., Machura B., Adamski S., Bozek O., Eksner B., Kokoszka B., Pekala T., Radom M., Strzelczak M. (2023). Deep learning automates bidimensional and volumetric tumor burden measurement from MRI in pre- and post-operative glioblastoma patients. Comput. Biol. Med..

[19] Zhang J., LaBella D., Zhang D., Houk J.L., Rudie J.D., Zou H., Warman P., Mazurowski M.A., Calabrese E. (2025). Development and evaluation of automated artificial intelligence–based brain tumor response assessment in patients with glioblastoma. AJNR Am. J. Neuroradiol..

[20] Suter Y., Notter M., Meier R., Loosli T., Schucht P., Wiest R., Reyes M., Knecht U. (2023). Evaluating automated longitudinal tumor measurements for glioblastoma response assessment. Front. Radiol..

[21] Shi T., Kujawa A., Linares C., Vercauteren T., Booth T.C. (2025). Automated longitudinal treatment response assessment of brain tumors: A systematic review. Neuro-Oncol..

[22] Bakas S., Vollmuth P., Galldiks N., Booth T.C., Aerts H.J.W.L., Bi W.L., Wiestler B., Tiwari P., Pati S., Baid U. (2024). Artificial intelligence for response assessment in neuro oncology (AI-RANO), part 2: Recommendations for standardisation, validation, and good clinical practice. Lancet Oncol..

[23] Villanueva-Meyer J.E., Bakas S., Tiwari P., Lupo J.M., Calabrese E., Davatzikos C., Bi W.L., Ismail M., Akbari H., Lohmann P. (2024). Artificial intelligence for response assessment in neuro oncology (AI-RANO), part 1: Review of current advancements. Lancet Oncol..

[24] Weinberg B.D., Gore A., Shu H.-K.G., Olson J.J., Duszak R., Voloschin A.D., Hoch M.J. (2018). Management-based structured reporting of posttreatment glioma response with the brain tumor reporting and data system. J. Am. Coll. Radiol..

[25] Gore A., Hoch M.J., Shu H.-K.G., Olson J.J., Voloschin A.D., Weinberg B.D. (2019). Institutional implementation of a structured reporting system: Our experience with the brain tumor reporting and data system. Acad. Radiol..

[26] Zhang J.Y., Weinberg B.D., Hu R., Saindane A., Mullins M., Allen J., Hoch M.J. (2020). Quantitative improvement in brain tumor MRI through structured reporting (BT-RADS). Acad. Radiol..

[27] Kujawa A., Shi T., Booth T., Vercauteren T. (2026). RANO2.0-assist: A 3D slicer extension for (semi-)automatic assessment of response to glioma treatment. J. Open Source Softw..

[28] Suter Y., Knecht U., Valenzuela W., Notter M., Hewer E., Schucht P., Wiest R., Reyes M. (2022). The LUMIERE dataset: Longitudinal glioblastoma MRI with expert RANO evaluation. Sci. Data.

[29] IBM Corp (2026). IBM SPSS Statistics for Windows.

[30] Python Software Foundation (2023). Python Language Reference.

[31] Mongan J., Moy L., Kahn C.E. (2020). Checklist for artificial intelligence in medical imaging (CLAIM): A guide for authors and reviewers. Radiol. Artif. Intell..

[32] Park S.H., Han K. (2018). Methodologic guide for evaluating clinical performance and effect of artificial intelligence technology for medical diagnosis and prediction. Radiology.

[33] Vaccaro M., Rosa E., Placidi E., Guarnera A., Secinaro A., Gandolfo C., Garganese M.C., Napolitano A. (2026). Deep learning for synthetic PET imaging: A systematic mapping review of techniques, metrics, and clinical relevance. Eur. Radiol. Exp..

