# Supplementary material for: Automated RANO 2.0-Aligned Response Assessment for Glioblastoma Clinical Trials: External Evaluation Against Expert Annotations
Source: Cancers (Basel). 2026 Oct 19;18(20):3383. doi: 10.3390/cancers18203383 (PMC13702278; doi:10.3390/cancers18203383)
Supplement: Supplementary file 1 [file cancers-18-03383-s001.zip › cancers-4603029-supplementary.pdf]

1. Supplementary Material

I) Supplementary Methods

Technical specification of the RANO-aligned longitudinal imaging-response pipeline

This supplementary section provides technical implementation details for the RANO-aligned longitudinal imaging-response pipeline underlying the automated tool described in the main manuscript. It is intended to make the criteria-execution layer auditable and reproducible, while avoiding duplication of the cohort definition, statistical analyses, and results reported in the main text. The pipeline converts serial MRI examinations and corresponding tumour segmentation masks into lesion entities, admissibility-filtered measurements, longitudinal lesion tracks, new-lesion flags, and provisional imaging-only response categories. Because corticosteroid exposure, neurological status, treatment context, and clinical deterioration are not incorporated, these outputs should be interpreted as imaging-only response categories rather than full clinical RANO 2.0 adjudications.

S1. Input data, study matching, and label definitions

The pipeline accepts volumetric MRI examinations and corresponding tumour segmentation masks in NIfTI format. Before analysis, the image and mask volumes undergo spatial-compatibility, voxel-dimension, and label-definition checks. Patient and study identifiers are parsed from filenames or study-level metadata and used to construct a unique patient-timepoint key. For the LUMIERE analysis, matching between pipeline outputs and expert annotations was performed using patient identifier and study date; only exact matches were retained.

The primary target label for the current analysis was contrast-enhancing tumour. Non-enhancing/necrotic tissue was used as an exclusion label for measurement-slice admissibility and did not contribute to enhancing tumour burden. No separate postoperative-cavity segmentation was generated. Other label sets, including non-enhancing T2/FLAIR abnormality, can be processed within the framework, but were not used as target burden inputs for the primary imaging-only response analyses reported in this study.

The input images and masks were not manually edited by the pipeline. Timepoints with segmentations judged to be clearly non-representative of tumour tissue were excluded at the analysis stage according to the predefined quality-control procedure described in the main manuscript.

S2. Lesion-entity definition and connectivity framework

Lesion definition is performed before any two-dimensional or volumetric measurement. A binary target mask is generated from the selected target label set, and lesion entities are defined from this mask using connected-component analysis. In the primary configuration, three-dimensional lesion separation is performed using 26-neighbour voxel connectivity. The software implementation also permits alternative three-dimensional connectivity definitions, including 6- and 18-neighbour connectivity, to support sensitivity testing or adaptation to different segmentation characteristics.

After connected-component separation, disconnected components may be merged into a single lesion entity when the physical separation between components is below a predefined distance threshold. Separation is calculated in physical units using voxel spacing, and the distance-based merging step is applied before measurement and longitudinal tracking. This allows spatially related enhancing foci to be treated as a single lesion entity under a prespecified configuration, while preserving the ability to audit how lesion-counting assumptions affect downstream outputs.

The framework also supports optional graph-based clustering of spatially proximate foci. In this mode, inter-focus distances are computed using boundary-to-boundary or centroid-based metrics, graph edges are formed for foci within the clustering threshold, and graph components satisfying a minimum foci-count criterion are grouped as cluster lesions. For clustered lesions, burden can be computed either from the union mask or, when enabled, by summing per-focus two-dimensional products to avoid spanning non-enhancing gaps between separated foci. These optional behaviours were used as configurable technical capabilities rather than as post hoc manual corrections.

S3. Slice admissibility and objective-driven slice selection

For each lesion entity, all axial slices intersecting the target mask are considered candidate measurement slices. A slice-admissibility rule is then applied to reduce the risk that bidimensional measurements are driven by necrotic, cystic, or postoperative-cavity components. In the current implementation, this rule quantifies the fraction of exclusion-label voxels within the lesion-derived two-dimensional measurement region on each candidate slice. Candidate slices exceeding the predefined exclusion threshold are rejected for measurement selection.

Among the remaining admissible slices, a method-specific objective function is computed, and the selected measurement slice is the admissible slice maximising that objective. For PPD, the objective is the axis-aligned perpendicular diameter product; for Feret-derived measurement, it is the Feret-derived product; for Max-PPD and Max-PPD-c, it is the corresponding optimised perpendicular-diameter product. This design separates lesion inclusion from slice selection: excluded slices are not used for diameter placement, but the lesion entity itself remains available for longitudinal tracking and response logic when evaluable.

For auditability, the implementation can export per-slice objective curves, selected-slice overlays, and neighbouring-slice montages. These outputs allow verification that the selected measurement slice was chosen by the prespecified objective within the admissible slice set.

S4. Bidimensional and volumetric measurement modules

The pipeline computes multiple tumour-burden representations within the same lesion-definition, slice-admissibility, and longitudinal framework. The implemented bidimensional methods are reported in the manuscript as PPD, Feret, Max-PPD, and Max-PPD-c. In code-level output tables, Max-PPD may appear as MaxProduct, and Max-PPD-c may appear as MaxFeretProductWithin; these names refer to the same manuscript-level metrics.

Classical axis-aligned perpendicular product (PPD) is calculated from the axis-aligned bounding-box extents of the lesion mask on the selected slice. The two perpendicular extents are converted to millimetres using voxel spacing, and their product is reported in mm^2. Feret-derived measurement uses caliper diameters across the two-dimensional lesion contour, deriving a major

caliper diameter and an associated perpendicular minor diameter, with the product reported as a Feret-derived area-like burden.

Max-PPD is an orientation-optimised maximum product of perpendicular diameters. Rather than restricting the measurement axes to image rows and columns, in-plane orientations are sampled, and perpendicular extents are calculated for each orientation. The retained metric is the maximum product across sampled orientations. This method provides an orientation-aware comparator to classical axis-aligned PPD, but does not impose the within-lesion chord constraint used by Max-PPD-c.

Max-PPD-c is the within-lesion chord-constrained maximum product of perpendicular diameters. For each candidate orientation on the selected slice, the algorithm samples candidate major chords within the lesion mask and identifies contiguous in-mask chord segments. Candidate intersection points are sampled along the major chord, and for each point a perpendicular in-mask minor chord is computed through the same point. The retained measurement is the maximum product of major and minor chord lengths across sampled orientations and intersection points. Both diameters are therefore constrained to lie entirely within contiguous enhancing tumour on the selected admissible slice. Measurement endpoints are stored for overlay-based audit.

Volumetric burden is calculated directly from the three-dimensional enhancing-tumour segmentation by multiplying the number of target-label voxels by voxel volume. Volume is handled as a three-dimensional contextual comparator and is not treated as a bidimensional SPD-equivalent measure.

S5. Lesion role assignment and tumour-burden aggregation

After measurement, lesion entities are assigned roles within a fixed RANO-aligned analytical configuration. Role assignment distinguishes target lesions, non-target lesions, non-measurable lesions, and excluded entities according to label class, measurability rules, and lesion-size criteria. In the present analysis, enhancing target lesions formed the basis for timepoint-level tumour-burden aggregation.

For each enabled measurement method, timepoint-level burden is calculated independently as the sum of the corresponding per-lesion target-burden values. Thus, PPD, Feret, Max-PPD, Max-PPD-c, and volumetric burden each generate separate timepoint-level burden outputs while sharing the same lesion definitions, slice-admissibility framework, longitudinal ordering, and response thresholds. This allows direct assessment of how measurement geometry influences response classification without changing lesion inputs.

When clustering or sum-of-foci analysis is enabled, burden aggregation follows the resulting lesion definition. For union-based cluster measurement, the clustered lesion is measured as a single lesion entity. For sum-of-foci analysis, the aggregate bidimensional burden is calculated as the sum of locally optimised per-focus products, avoiding artificial inflation across non-enhancing inter-focus gaps.

S6. Longitudinal ordering, TrackID propagation, and new-lesion logic

Timepoints were ordered longitudinally for each patient using the relative study dates provided in LUMIERE. The earliest available examination was used to initialise longitudinal lesion tracking, and each lesion at this timepoint was assigned a deterministic lesion identifier (TrackID). The earliest

post-operative examination was used as the response-assessment baseline, as described in Section S8. Its timing relative to radiotherapy completion was not verified.

At follow-up, lesion entities are matched to prior TrackIDs using a hierarchical correspondence strategy. The primary correspondence criterion is spatial overlap; if overlap is insufficient or absent, distance-based correspondence can be evaluated using boundary-to-boundary and/or centroid distances in physical units. Matching prioritises overlap-based correspondence over distance-based correspondence. Matched lesions inherit the prior TrackID, whereas unmatched lesions are assigned new TrackIDs and flagged as new relative to the immediately preceding timepoint.

A lesion absent at an intervening timepoint but subsequently matched to an earlier TrackID is flagged as reappeared. Reappeared lesions can be retained as reappeared states or treated as new for downstream progression logic according to configuration. Baseline target-lesion identifiers can be propagated through TrackID linkage, enabling follow-up measurements to be audited against the same tracked target lesion rather than against a purely timepoint-local lesion number.

S7. Optional registration and anatomical contextualisation

The longitudinal framework can operate with or without inter-scan registration. When serial masks and images are already provided in a compatible spatial frame, tracking can proceed directly in native voxel coordinates after dimensional and alignment checks. When registration is enabled, a selected reference image can be aligned to the patient image space using rigid or affine transformation, and correspondence can be evaluated in the resulting spatial frame. If image geometry is incompatible and no valid correspondence can be established, tracking outputs are not forced. Registration quality was not independently assessed for individual discordant new-lesion calls.

An optional anatomical contextualisation module can append atlas- or parcellation-derived anatomical labels to lesion outputs. This module is descriptive only: it does not modify lesion separation, measurement selection, burden aggregation, longitudinal tracking, or response classification. It is intended for audit and reporting rather than for response adjudication.

S8. Imaging-based RANO response module

Response categories are assigned after per-lesion measurements have been aggregated into timepoint-level tumour-burden outputs. Calculations are performed separately for each enabled measurement method, allowing response labels derived from PPD, Feret, Max-PPD, Max-PPD-c, and volumetric burden to be compared under identical lesion-definition and longitudinal assumptions.

In the current RANO-aligned imaging-only implementation, response labels are generated by applying deterministic quantitative rules to segmentation-derived enhancing-tumour burden and longitudinal lesion-state flags. Complete response is assigned when the summed measurements of the selected enhancing target lesions are zero. Partial response requires a ≥50% decrease in bidimensional burden or a ≥65% decrease in volumetric burden according to the configured longitudinal reference rule and without a pipeline-defined new enhancing lesion. Progressive disease is assigned for a ≥25% increase in bidimensional burden, a ≥40% increase in volumetric burden, or a new enhancing lesion identified by TrackID-based longitudinal matching. A pipeline-defined new enhancing lesion overrides burden-based response categories and results in PD. Stable disease is assigned when none of these criteria are met. These rules are applied separately

for each measurement method under the same lesion-definition, tracking, and aggregation framework. The implementation is therefore an imaging-only criteria-execution module and does not include the clinical, treatment-context, confirmation, steroid, neurological, or non-enhancing-disease components required for full RANO 2.0 adjudication.

For the present LUMIERE analysis, the earliest post-operative MRI in each patient's series was used as the response-assessment baseline. A first post-radiotherapy baseline was not provided in the dataset and therefore could not be verified. PD thresholds were evaluated relative to a positive baseline burden or a positive prior nadir. The outputs were interpreted as provisional RANO 2.0-aligned imaging-only response categories.

S9. Quality-control outputs and audit trail

A central design goal was auditability. The pipeline exports a timepoint-level summary table and a lesion-level detail table containing lesion identifiers, lesion roles, measurement values, selected slices, measurement endpoints, TrackIDs, new/reappeared lesion states, and response outputs where applicable. These tables provide the link between segmentation-derived lesion entities and final timepoint-level response labels.

Image-based audit outputs can include selected-slice overlays showing lesion masks and measurement line segments, montages centred on selected measurement slices, per-slice objective score curves, time-series plots of tumour burden, and post hoc overlays annotated with TrackIDs and response labels. Optional spatial outputs, including QC mask volumes, preserve the spatial reference information of the source image to support viewer alignment.

The audit outputs were generated to support reproducibility and failure-mode analysis. They were not used to manually correct measurements or reassign response categories after pipeline execution.

S10. Reproducibility constraints and interpretation

The primary analyses were performed under a fixed analytical configuration so that comparisons between measurement methods were not confounded by changes in lesion definition, target-lesion selection, slice admissibility, longitudinal ordering, burden aggregation, or response thresholds. Alternative measurement geometries were therefore evaluated as method-level variants within the same automated imaging response assessment tool rather than as separate pipelines.

The framework is deterministic for a given set of inputs and configuration parameters. Any exclusion of timepoints was performed at the analysis stage according to predefined quality-control criteria and did not involve manual correction of source images or segmentation masks. The resulting outputs are intended for technical evaluation, decision support, and research standardisation, not autonomous clinical adjudication.

S11. Pipeline configuration

For the primary analysis, all numeric configuration parameters used were applied uniformly across all patients and timepoints. These included lesion-separation distance (5 mm), slice-admissibility exclusion threshold (5%), minimum measurable lesion size (10 mm for target lesions), longitudinal tracking tolerance (3 mm), and new-lesion overlap threshold (1%). Additional parameters related to clustering, non-target lesion thresholds, and optional processing modules were included in the same configuration but were not activated in the primary analysis. No parameter was manually

adjusted based on inspection of expert measurements. Within each examination, lesion-definition and processing settings were shared across measurement methods. The thresholds, parameters and response decision rules used in the reported comparisons were fixed before examining performance against the LUMIERE expert annotations.

S12. Metric terminology

Term	Definition
**PPD**	Axis-aligned product of perpendicular diameters on the selected slice.
**Feret**	Calliper-derived major diameter and associated perpendicular minor diameter across the lesion contour.
**Max-PPD**	Orientation-optimised maximum product of perpendicular diameters without the within-lesion chord constraint.
**Max-PPD-c**	Within-lesion chord-constrained maximum product of perpendicular diameters; both diameters remain inside contiguous enhancing tumour.
**Volume**	Three-dimensional target-label volume derived from the segmentation mask.

II) Supplementary Figures

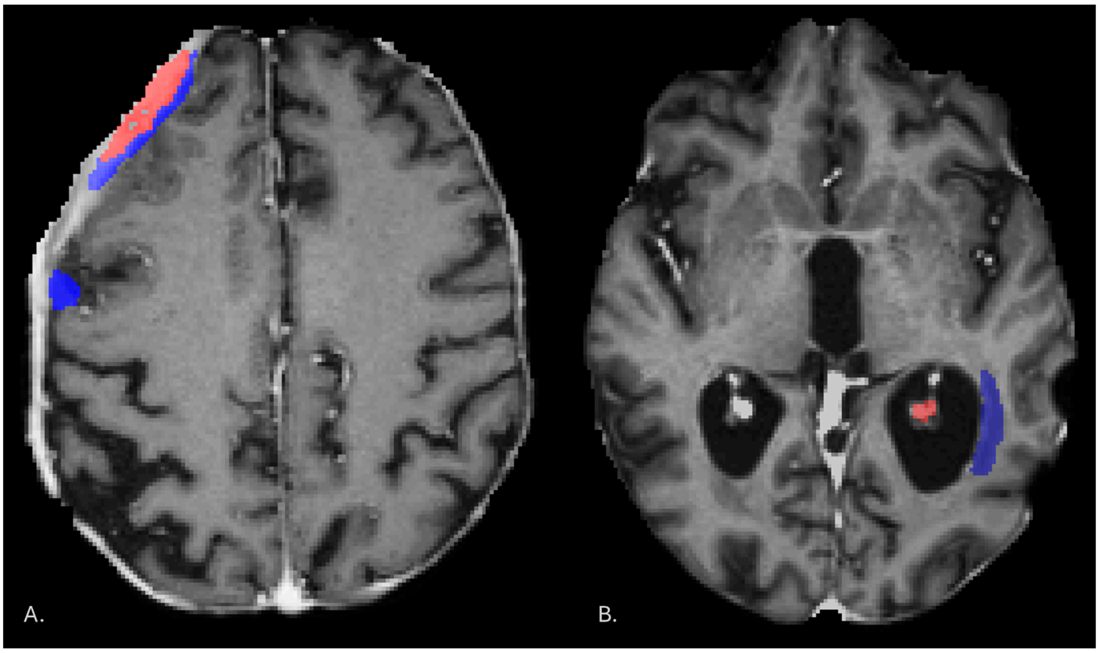

Figure S1. Examples of segmentation quality-control exclusions.

Representative axial post-contrast T1-weighted images with overlaid dataset-provided segmentation masks are shown for examinations excluded by the prespecified quality-control procedure. (A) Extra-axial/subdural collection incorrectly labelled as enhancing tumour. (B) Choroid plexus enhancement incorrectly segmented as tumour tissue. Quality-control review was performed before final concordance analyses by an expert neuroradiologist blinded to expert RANO category, expert bidimensional measurements, progression timing, and downstream pipeline-expert agreement results. No source image or segmentation mask was manually edited.

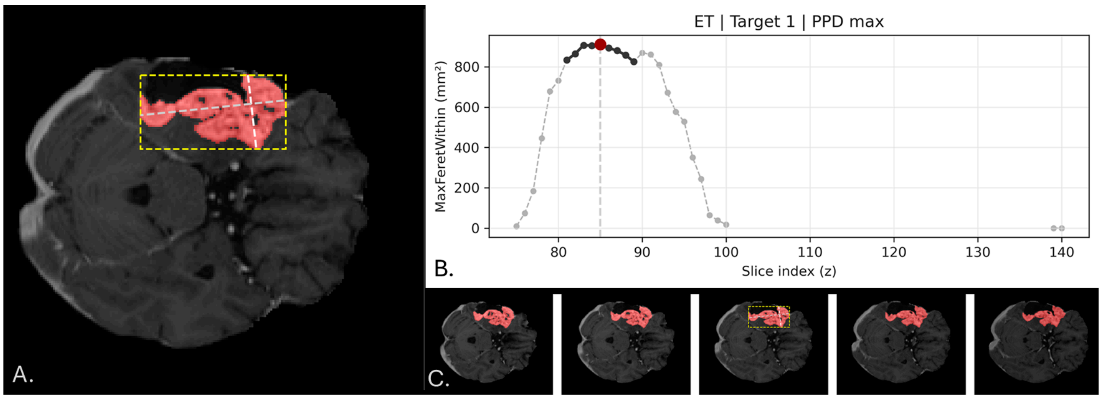

Figure S2. Example of auditable Max-PPD-c measurement and slice selection.

Representative post-contrast T1-weighted images show a cavity-adjacent enhancing lesion processed by the deterministic measurement module.
(A) Selected measurement slice with enhancing-tumour overlay and Max-PPD-c measurement chords. (B) Slice-selection objective curve showing the prespecified measurement objective across candidate slices, with the selected slice highlighted. (C) Neighbouring-slice montage illustrating the audit trail used to verify slice selection and measurement placement.
The selected Max-PPD-c slice was chosen from admissible candidate slices according to the predefined objective function. Measurement chords were constrained to remain within contiguous enhancing tumour, while slices exceeding the predefined necrotic or cavity-label threshold were excluded from measurement selection. Max-PPD-c: within-lesion chord-constrained maximum product of perpendicular diameters.

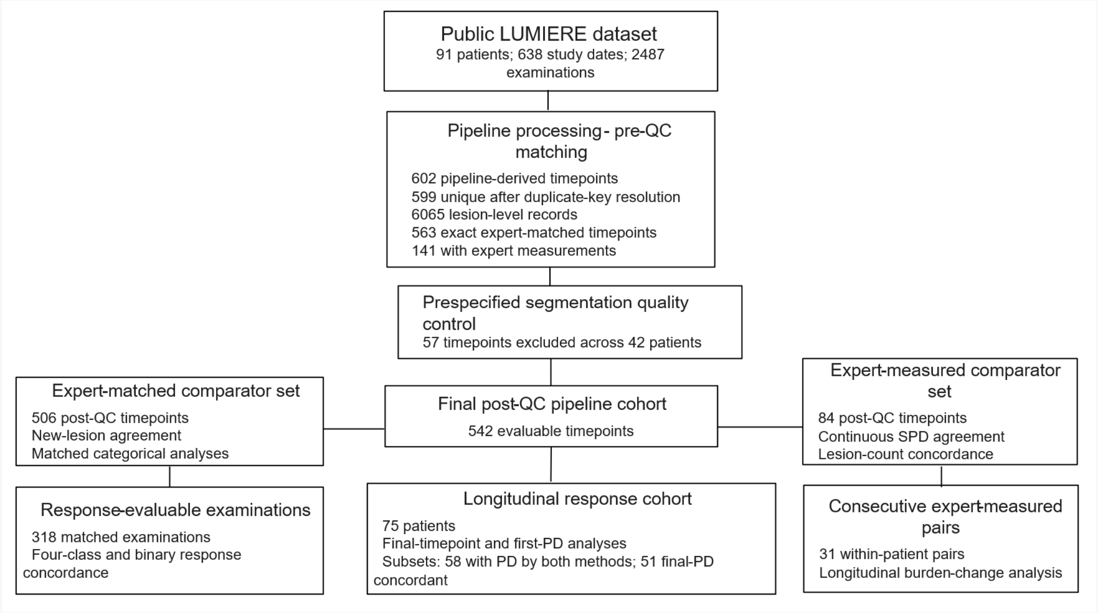

Figure S3. Cohort flow and analysis populations.
The public LUMIERE dataset comprised 91 patients, 638 study dates, and 2487 examinations. The deterministic framework generated 602 pipeline-derived timepoints, of which 599 were unique after duplicate-key resolution. Before quality control, 563 timepoints matched expert RANO imaging annotations and 141 had expert bidimensional measurements. Prespecified blinded neuroradiologist QC excluded 57 timepoints across 42 patients because the dataset-provided segmentation was clearly non-representative of tumour tissue; no image or mask was edited. The final post-QC cohort included 542 evaluable timepoints, including 506 expert-matched timepoints, 84 expert-measured timepoints, 318 response-evaluable matched examinations, 31 consecutive expert-measured pairs, and 75 patients in longitudinal response analyses. QC=quality control; SPD=sum of products of diameters; PD=progressive disease.

III) Supplementary Tables

Table S1. Summary of automated pipeline performance against expert RANO assessment.

Domain	Analysis set	Metric	Result
Internal method consistency	318 examinations	Automated CR/PR/SD/PD agreement across methods	100.0%; κ=1.00
Primary SPD agreement	84 expert-measured timepoints	Max-PPD-c vs expert SPD Pearson r / Spearman ρ	0.753 / 0.861
Primary SPD agreement	84 expert-measured timepoints	Median absolute percentage difference	36.8%
Primary SPD agreement	84 expert-measured timepoints	Within ±50% / ±100% of expert SPD	73.8% / 94.0%
Primary SPD agreement	84 expert-measured timepoints	Bland-Altman bias; LoA, mm²	-106.8; -1185.0 to 971.5
Longitudinal burden change	31 consecutive expert-measured pairs	Percentage-change Pearson r / Spearman ρ	0.715 / 0.874
Longitudinal burden change	31 consecutive expert-measured pairs	Directional / clinical-threshold agreement	77.4% / 71.0%
Per-timepoint response concordance	318 matched examinations	Four-class CR/PR/SD/PD exact agreement; κ	48.1%; κ=0.173
Binary PD/non-PD concordance	318 matched examinations	Observed agreement; κ	64.2%; κ=0.263
Binary PD/non-PD concordance	318 matched examinations	Sensitivity / specificity	69.2% / 57.1%
Binary PD/non-PD concordance	318 matched examinations	PPV / NPV	69.2% / 57.1%
Final-timepoint response concordance	75 patients	Four-class / binary agreement	69.3% / 76.0%
First-PD timing categories	75 patients	Same / earlier pipeline / later pipeline / no PD by one or both	34.7% / 28.0% / 14.7% / 22.7%
Paired first-PD timing	58 patients with PD by both methods	Same / earlier pipeline / later pipeline; median Δweek	44.8% / 36.2% / 19.0%; 0.0 weeks
Concordant final-PD subgroup	51 patients	Same / earlier pipeline / later pipeline; median Δweek	43.1% / 39.2% / 17.6%; 0.0 weeks
New-lesion concordance	506 expert-matched timepoints	Exact agreement; κ	55.9%; κ=0.114
New-lesion concordance	506 expert-matched timepoints	Sensitivity / specificity	66.7% / 53.8%
New-lesion concordance	506 expert-matched timepoints	PPV / NPV	22.3% / 89.0%
New-lesion concordance counts	506 expert-matched timepoints	Concordant positive / concordant negative / pipeline-only positive / expert-only positive	56 / 227 / 195 / 28
Lesion-count concordance	84 expert-measured timepoints	Exact count agreement; pipeline more / fewer lesions	75.0%; 8 / 13

Values are reported at the examination/timepoint level unless the analysis set explicitly states patients. Confidence intervals were calculated using patient-level clustered bootstrap resampling. The primary automated bidimensional metric was Max-PPD-c. Expert RANO imaging annotations and measurements were used as comparators and should not be interpreted as final clinical RANO 2.0 adjudications. Binary and new-lesion measures are descriptive concordance measures, not independent diagnostic-performance estimates. Bland-Altman values are pipeline minus expert SPD. Δweek was defined as pipeline first-PD week minus expert first-PD week; negative values indicate earlier pipeline PD assignment. CR=complete response; LoA=limits of agreement; NPV=negative predictive value; PD=progressive disease; PPV=positive predictive value; PR=partial response; SD=stable disease; SPD=sum of products of diameters.

Table S2. Exploratory comparison of automated bidimensional tumour-burden methods against expert SPD.

Method	Pearson r (95% CI)	Spearman ρ (95% CI)	CCC (95% CI)	Median absolute percentage difference (95% CI)	Within ±50% of expert SPD (95% CI)	Bland–Altman LoA width, mm² (95% CI)
**Max-PPD-c (primary)**	0.753 (0.668–0.835)	0.861 (0.781–0.906)	0.680 (0.575–0.808)	36.8% (28.9–41.5%)	62/84; 73.8% (65.8–81.3%)	2156.5 (1454.6 - 2649.5)
**Feret-derived PPD**	0.747 (0.581–0.881)	0.788 (0.636–0.877)	0.726 (0.571–0.791)	29.7% (23.9–41.5%)	57/84; 67.9% (58.3–76.0%)	2703.2 (1848.3 - 3528.4)
**Axis-aligned PPD**	0.754 (0.569–0.897)	0.797 (0.646–0.892)	0.719 (0.535–0.800)	33.7% (25.2–44.1%)	56/84; 66.7% (55.3–75.7%)	2790.0 (1761.8 to 3672.6)
**Max-PPD**	0.771 (0.615–0.902)	0.810 (0.669–0.889)	0.669 (0.534–0.751)	40.2% (31.6–47.2%)	53/84; 63.1% (52.2–73.4%)	3277.5 (2103.6 - 4342.5)

Max-PPD-c had the highest Spearman correlation, the highest proportion of observations within ±50% of expert SPD and the narrowest Bland–Altman limits among the tested methods. CCC=concordance correlation coefficient; LoA=limits of agreement; PPD=perpendicular product of diameters; SPD=sum of products of diameters.

Exploratory temporal cross-confirmation of discordant new-lesion calls

Exploratory temporal cross-confirmation analyses assessed whether discordant new-lesion calls by one method were followed by subsequent positivity of the other method at the next available follow-up, within prespecified ≤12-week and ≤24-week windows, or at any later follow-up. Analyses used the first discordant event per patient per direction to reduce inflation from repeated longitudinal calls. Because expert and automated lesion identifiers were not shared, analyses assessed patient-level temporal concordance rather than lesion-level confirmation.

Direction-specific and combined temporal cross-confirmation analyses are reported in Table S3, whereas conservative all-index temporal confirmation analyses are reported in Table S4. Pipeline-leading discordances showed lower short-window confirmation than expert-leading discordances, although only 11 expert-leading index events were available.

Exploratory temporal analyses demonstrated partial later cross-confirmation of discordant new-lesion calls. In the combined bidirectional analysis, cross-confirmation occurred in 27.4% of evaluable discordant events at the next follow-up, 33.3% within 12 weeks, 36.4% within 24 weeks, and 61.3% at any later follow-up. These findings support partial temporal convergence of discordant new-lesion calls at patient level but do not establish same-lesion confirmation.

Table S3. Bidirectional temporal cross-confirmation of discordant new-lesion calls.

Direction	Window	Index events	Evaluable	Confirmed	Confirmation rate	95% CI
**Pipeline+/Expert−**	Next visit	63	52	11	21.2%	10.4 - 32.7
**Pipeline+/Expert−**	≤12 weeks	63	18	3	16.7%	0.0 - 35.7
**Pipeline+/Expert−**	≤24 weeks	63	46	14	30.4%	17.3 - 44.2
**Pipeline+/Expert−**	Any later	63	52	29	55.8%	42.0 - 69.1
**Expert+/Pipeline−**	Next visit	11	10	6	60.0%	25.0 - 90.0
**Expert+/Pipeline−**	≤12 weeks	11	6	5	83.3%	50.0 - 100.0
**Expert+/Pipeline−**	≤24 weeks	11	9	6	66.7%	33.3 - 100.0
**Expert+/Pipeline−**	Any later	11	10	9	90.0%	66.7 - 100.0
**Combined**	Next visit	74	62	17	27.4%	16.9 - 39.1
**Combined**	≤12 weeks	74	24	8	33.3%	14.3 - 52.9
**Combined**	≤24 weeks	74	55	20	36.4%	23.5 - 49.2
**Combined**	Any later	74	62	38	61.3%	49.2 - 73.2

Exploratory analyses assessed whether discordant new-lesion calls by one method were followed by subsequent positivity of the other method at the next available follow-up, within prespecified ≤12-week and ≤24-week windows, or at any later follow-up. Analyses used the first discordant event per patient per direction to reduce inflation from repeated longitudinal calls. Combined

analyses pooled pipeline-leading and expert-leading discordances. Because expert and automated lesion identifiers were not shared, confirmation represents patient-level temporal concordance rather than lesion-level verification. Confidence intervals were calculated using patient-level clustered bootstrap resampling. To provide lower-bound temporal confirmation estimates independent of window evaluability, conservative all-index analyses were also performed.

Table S4. Conservative all-index temporal confirmation analysis of discordant new-lesion calls.

**Direction**	**≤12 weeks**	**≤24 weeks**
**Pipeline-leading**	3/63 (4.8%)	14/63 (22.2%)
**Expert-leading**	5/11 (45.5%)	6/11 (54.5%)
**Combined**	8/74 (10.8%)	20/74 (27.0%)

Conservative analyses used all discordant index events as denominators, including events without eligible follow-up within the specified temporal window. Values therefore represent lower-bound temporal confirmation estimates. Combined analyses pooled pipeline-leading and expert-leading discordances. Because expert and automated lesion identifiers were not shared, analyses represent patient-level temporal concordance rather than lesion-level confirmation.

Table S5. A. Agreement and disagreement across measurement methods.

Method	Post-QC disagreements	Post-QC agreements	Pre-QC disagreements
**PPD**	14/31 (45.2%)	17/31 (54.8%)	36/65 (55.4%)
**Feret**	11/31 (35.5%)	20/31 (64.5%)	32/65 (49.2%)
**Max-PPD**	13/31 (41.9%)	18/31 (58.1%)	35/65 (53.8%)
**Max-PPD-c**	9/31 (29.0%)	22/31 (71.0%)	33/65 (50.8%)

Table S5. B. Expert versus automated burden-threshold classifications for Max-PPD-c after QC. Rows represent expert categories and columns represent automated categories. Agreement occurred in 22/31 pairs (71.0%) and disagreement in 9/31 pairs (29.0%).

Expert category	Automated improving	Automated stable	Automated worsening
**Improving**	2	2	0
**Stable**	1	5	1
**Worsening**	0	5	15

Table S5: Percentage changes between consecutive examinations were classified as improving (≥50% decrease), worsening (≥25% increase) or stable between these thresholds. Disagreement occurred when expert and automated measurements assigned different categories to the same examination pair. After QC, the analysis included 31 pairs from 17 patients. Before QC, 65 pairs from 33 patients had defined percentage changes. Fourteen of the 79 candidate pairs had undefined automated percentage changes because the previous automated burden was zero and were excluded from this calculation. These categories describe consecutive-pair burden changes rather than complete RANO response categories.

Table S6. Performance before and after visual segmentation quality control.

A. Cohort sizes and endpoint-specific exclusions

Endpoint	Before QC timepoints / patients	After QC timepoints / patients	Patients with complete / partial exclusions
**All pipeline timepoints**	599 / 91	542 / 91	0 / 42
**Expert-matched timepoints**	563 / 91	506 / 91	0 / 42
**Expert SPD comparator**	141 / 62	84 / 47	15 / 27
**Categorical response**	375 / 79	318 / 75	4 / 38

B. Continuous SPD comparisons across measurement methods

Method	QC status	n	Pearson r	Spearman ρ	CCC	Median absolute percentage difference (%)
**PPD**	Before QC	141	0.383	0.475	0.378	62.5%
**PPD**	After QC	84	0.754	0.797	0.719	33.7%
**Feret**	Before QC	141	0.427	0.455	0.417	63.5%
**Feret**	After QC	84	0.747	0.788	0.726	29.7%
**Max-PPD**	Before QC	141	0.370	0.481	0.351	70.8%
**Max-PPD**	After QC	84	0.771	0.810	0.668	40.2%
**Max-PPD-c**	Before QC	141	0.336	0.397	0.269	63.5%
**Max-PPD-c**	After QC	84	0.753	0.861	0.680	36.8%

C. Max-PPD-c Bland–Altman results and tolerance agreement

Measure	Before QC	After QC
**Mean pipeline minus expert SPD (mm²)**	−352.53	−106.75
**Bland–Altman limits of agreement (mm²)**	−2230.39 to 1525.33	−1185.02 to 971.52
**Within ±50% of expert SPD**	62/141 (44.0%)	62/84 (73.8%)

D. Categorical response and new-lesion agreement

Measure	Before QC	After QC
**Response exact four-class agreement**	203/375 (54.1%)	153/318 (48.1%)
**Response unweighted four-class κ**	0.196	0.173
**Response binary PD agreement**	254/375 (67.7%)	204/318 (64.2%)
**Response PD sensitivity**	75.4%	69.2%
**Response non-PD specificity**	54.7%	57.1%

**New-lesion examinations**	563	506
**New-lesion TP / TN / FP / FN**	77 / 228 / 230 / 28	56 / 227 / 195 / 28
**New-lesion sensitivity / specificity**	73.3% / 49.8%	66.7% / 53.8%
**New-lesion PPV / NPV**	25.1% / 89.1%	22.3% / 89.0%
**New-lesion exact binary agreement**	54.2%	55.9%
**New-lesion κ**	0.133	0.114
**Final-visit exact four-class agreement**	62/79 (78.5%)	52/75 (69.3%)
**Final-visit binary PD agreement**	65/79 (82.3%)	57/75 (76.0%)

Visual segmentation QC excluded 57 timepoints across 42 patients when an expert neuroradiologist confirmed that the dataset-provided masks were clearly non-representative of tumour tissue. Source images and masks were not edited. Complete and partial exclusions are specific to each endpoint, and the endpoint populations overlap. All 91 patients retained at least one pipeline timepoint. SPD is expressed in mm^2, and the median absolute percentage difference is relative to expert SPD. Expert imaging annotations served as the comparator for response and new-lesion agreement. Categorical outputs are provisional RANO 2.0-aligned imaging-only response categories. Final examinations were selected separately as the last eligible examination before and after QC, so final-timepoint comparisons may reflect changes in visit selection as well as patient eligibility. QC denotes quality control, SPD sum of products of diameters, CCC concordance correlation coefficient and PD progressive disease. PPV and NPV denote positive and negative predictive value, respectively. TP, TN, FP and FN denote true-positive, true-negative, false-positive and false-negative findings, respectively, relative to the expert annotations.

Table S7. Response-rule flow and effect of suppressing the new-lesion override.

A. Response-rule flow in 318 examinations

Category	Assigning rule	Examinations, n (%)
PD	New-lesion override	185 (58.2%)
CR	Summed target-lesion measurements were greater than zero at baseline and zero at follow-up	101 (31.8%)
CR	Summed target-lesion measurements were zero at both baseline and follow-up	32 (10.1%)

B. Override suppression in 318 examinations

Method	Changed categories, n	Four-class agreement, n (%)	Unweighted κ	CR / PR / SD / PD, n
PPD	125	82/318 (25.8%)	0.076	194/27/37/60
Feret	124	85/318 (26.7%)	0.090	194/31/32/61
Max-PPD	122	85/318 (26.7%)	0.084	194/26/35/63
Max-PPD-c	123	84/318 (26.4%)	0.087	194/34/28/62
Volume	140	65/318 (20.4%)	0.043	194/47/32/45

C. Override suppression at 75 final visits

Method	Changed categories, n	Four-class agreement, n (%)	Binary PD agreement, n (%)
PPD	29	29/75 (38.7%)	38/75 (50.7%)
Feret	29	29/75 (38.7%)	38/75 (50.7%)
Max-PPD	29	29/75 (38.7%)	38/75 (50.7%)
Max-PPD-c	30	27/75 (36.0%)	37/75 (49.3%)
Volume	39	19/75 (25.3%)	28/75 (37.3%)

D. Effect of removing the new-lesion override on agreement with expert categories across measurement methods

Effect of removing the override	PPD	Feret	Max-PPD	Max-PPD-c	Volume
Previously disagreed with the expert category and now agreed	8 (2.5%)	9 (2.8%)	8 (2.5%)	8 (2.5%)	5 (1.6%)
Previously disagreed with the expert category and still disagreed, despite changing category	38 (11.9%)	38 (11.9%)	38 (11.9%)	38 (11.9%)	42 (13.2%)
Previously agreed with the expert category and now disagreed	79 (24.8%)	77 (24.2%)	76 (23.9%)	77 (24.2%)	93 (29.2%)
**Total examinations with a changed pipeline category**	**125 (39.3%)**	**124 (39.0%)**	**122 (38.4%)**	**123 (38.7%)**	**140 (44.0%)**

Panel A reports the rule that assigned each response category. The same counts applied to all five measurement methods. CR based on zero summed measurements of the selected target lesions did not establish disappearance of all enhancing disease.

Panels B and C report results after removing only the new-lesion override, using the same saved measurements, baseline, prior nadir and response-rule order. The comparisons used the same 318 response-evaluable examinations from 75 patients and the same 75 selected final visits, one per patient. These final visits differ from the separately selected visits used for the before-and-after QC comparison in Table S6. With the override, all methods had four-class agreement of 153/318 (48.1%) and unweighted Cohen's κ of 0.173. At the final visits, four-class agreement was 52/75 (69.3%) and binary PD agreement was 57/75 (76.0%). Four-class agreement compares CR, PR, SD and PD with expert imaging annotations. Binary PD agreement compares PD with non-PD.

Panel D reports the number of examinations whose category changed after removing the override for each measurement method. For each method, the three outcome rows are mutually exclusive and their counts sum to its total number of changed categories. Percentages in Panel D use all 318 examinations as the denominator for each method. Unchanged categories are not included in Panel D. Agreement and disagreement refer to the expert category at the same examination. The comparison assesses the effect of the override on response categories and does not determine whether individual new-lesion flags were correct. CR=complete response; PR=partial response; SD=stable disease; PD=progressive disease; QC=quality control.

Table S8. Expert–pipeline response confusion matrices and agreement estimates.

A. 318 response-evaluable examinations from 75 patients

Expert category	Pipeline CR	Pipeline PR	Pipeline SD	Pipeline PD	Expert total
CR	25	0	0	1	26
PR	8	0	0	11	19
SD	43	0	0	45	88
PD	57	0	0	128	185
**Pipeline total**	**133**	**0**	**0**	**185**	**318**

B. Final visits in 75 patients

Expert category	Pipeline CR	Pipeline PR	Pipeline SD	Pipeline PD	Expert total
CR	1	0	0	0	1
PR	4	0	0	1	5
SD	1	0	0	6	7
PD	11	0	0	51	62
**Pipeline total**	**17**	**0**	**0**	**58**	**75**

C. Agreement estimates and 95% confidence intervals

Metric	318 examinations		75 final visits	
	Estimate	95% CI	Estimate	95% CI
Exact agreement, %	48.1	41.4–54.3	69.3	58.7–78.7
Binary PD agreement, %	64.2	59.2–68.9	76.0	66.7–85.3
Unweighted κ	0.173	0.094–0.252	0.143	0.008–0.284
Linear weighted κ	0.225	0.120–0.331	0.241	0.051–0.430
Quadratic weighted κ	0.270	0.148–0.393	0.277	0.057–0.503

Values are examination counts, with expert imaging categories in rows and pipeline categories in columns. All five measurement methods produced identical matrices and agreement estimates. Panel B includes one selected final visit per patient from panel A. Exact agreement compares four-class categories. Binary PD agreement compares PD with non-PD (CR, PR or SD). Unweighted Cohen's κ was primary. Linear and quadratic weighted κ were exploratory, with categories ordered as CR, PR, SD and PD, indexed 0–3. Linear disagreement weights were $\frac{|i-j|}{3}$ and quadratic weights were $\frac{(i-j)^2}{9}$. Confidence intervals used 2000 patient-level clustered percentile bootstrap replicates, retaining all examinations of each resampled patient. Final visits were selected before resampling. All 2000 replicates were valid for each metric. Pipeline categories are provisional RANO 2.0-aligned imaging-only response categories.

CI, confidence interval. CR, complete response. PR, partial response. SD, stable disease. PD, progressive disease.

Table S9. Frequencies of observed discrepancies and matching findings.

Observation	n/N (%)	Description
Higher pipeline measurements in QC-excluded examinations	10/57 (17.5%)	Max-PPD-c measurements were higher than expert SPD.
Lower pipeline measurements in QC-excluded examinations	47/57 (82.5%)	Max-PPD-c measurements were lower than expert SPD. These included 27 examinations with zero summed target-lesion measurements and positive expert SPD.
Target-lesion count disagreement	21/84 (25.0%)	The pipeline count was higher in 8 examinations and lower in 13.
Max-PPD-c burden-threshold disagreement	9/31 (29.0%)	Pipeline and expert measurements gave different threshold categories for consecutive examination pairs from 17 patients.
Pipeline-only new-lesion positivity	195/506 (38.5%)	Pipeline-positive and expert-negative new-lesion annotations at the same examination.
Expert-only new-lesion positivity	28/506 (5.5%)	Expert-positive and pipeline-negative new-lesion annotations at the same examination.
Four-class response disagreement	165/318 (51.9%)	The pipeline and expert CR, PR, SD or PD categories differed.
PD assigned by the new-lesion override with an expert non-PD category	57/165 (34.5%)	Of the 165 discordant categories, 57 were override-generated PD assignments with an expert CR, PR or SD category.
CR assignments with non-target lesions recorded	19/133 (14.3%)	Non-target enhancing lesions were recorded despite zero summed target-lesion measurements.
Lesion measurement records with unmatched detection components	6065/6065 (100.0%)	Component-match flags were false and detection-component TrackIDs were null.
Follow-up examinations with new-component count equal to total detected-component count	508/508 (100.0%)	The reported number of new enhancing detection components equalled the total number of detected enhancing components.

Table S9 summarises the observed measurement discrepancies, response disagreements and longitudinal matching findings. Values are presented as n/N (%), using the denominator relevant to each comparison. Observations may overlap. Among the 57 examinations excluded following visual segmentation QC, pipeline measurements were higher than expert SPD in 10 and lower in 47. Measurement disagreement alone was not an exclusion criterion.

Measurement and burden-threshold comparisons refer to Max-PPD-c. Threshold comparisons used ≥25% increase and ≥50% reduction between consecutive examinations. The response counts were identical across all five measurement methods. These comparisons used the post-QC analysis sets. Component-mapping findings were examined before QC, using all 6065 lesion measurement records after duplicate resolution and all 508 follow-up examinations. Separate measurement records were available for each method. The relative contributions of registration, segmentation, postsurgical change and lesion splitting or merging were not independently adjudicated.

CR=complete response; PD=progressive disease; PR=partial response; QC=quality control; SD=stable disease; SPD=sum of products of diameters.
